# Evolution of crystallographic texture and material anisotropy effects resulting from uniaxial deformation for high-strength steels with high manganese content

**DOI:** 10.1107/S1600576725010350

**Published:** 2026-02-01

**Authors:** Michael Zuern, Morteza Dadkhah, Thomas Nitschke-Pagel, Jens Gibmeier

**Affiliations:** a Institute for Applied Materials (IAM-WK) Karlsruhe Institute of Technology Engelbert-Arnold-Straße 4 76131 Karlsruhe Germany; b Institute of Joining and Welding (IFS) TU Braunschweig Langer Kamp 8 38106 Braunschweig Germany; Montanuniversität Leoben, Austria

**Keywords:** TRIP/TWIP steels, elastic anisotropy, crystallographic texture, intergranular strains

## Abstract

Steels used in the automotive industry have seen significant improvements within the past few decades, as economic and environmental efficiency play major and increasing roles in current individual transportation. In particular, it has been possible to combine high formability with high strength, which contributes to passenger safety, vehicle performance and efficiency. Deep-drawing steels with higher manganese content play a special role in the class of automotive body sheet materials, in which the final mechanical properties are achieved, for example, through twinning-induced plasticity and/or transformation-induced plasticity effects. In this study, the deformation behavior of steels X40MnCrAlV-19-2.5-2, HCT690T and S355MC was investigated using laboratory X-ray diffraction analyses. The investigations cover the evolution of crystallographic texture and material anisotropy resulting from uniaxial deformation, as well as their influence on diffraction data, especially in the context of stress analyses. In contrast to the situation in the initial as-rolled state, the induced deformations up to the limit of uniform elongation lead to the formation of strong textures and a considerable impairment of the diffraction data due to material anisotropy. However, the formed crystallographic textures do not suffice to describe this impairment. Rather, this effect is mainly attributable to intergranular strains, which are caused by different degrees of deformation of individual crystallites within the elasto-plastic regime. These phenomena need to be considered, and this is demonstrated using the application example of a welded metal sheet. Correction approaches are proposed and their application is illustrated, with a focus on how readily the residual stresses can be evaluated.

## Abstract 2

The present study investigates the deformation behavior of high-strength steels and the associated evolution of material anisotropy. Particular focus is placed on the impact of crystallographic texture and intergranular strains on how residual stresses can be evaluated using X-ray diffraction and the associated assessment of weld seams in the automotive industry.

## 1. Introduction

Efficiency and lightweight construction continue to play central roles in automotive engineering alongside electrification, both economically and in terms of environmental policy. For this reason, the use of a wide variety of steels and non-ferrous metals is now state of the art. Steels are still important in vehicle construction, as it has been possible to combine high formability with high strength. These high-strength and ultra-high-strength deep-drawing steels are used particularly in car body construction and lead to increased occupant safety, as well as improved vehicle performance and efficiency. Steels with a high manganese content play a special role here (Hilditch *et al.*, 2015 ▸; De Cooman *et al.*, 2011 ▸; Cornette *et al.*, 2001 ▸; Cornette *et al.*, 2005 ▸; Fonstein, 2015 ▸; Weidner, 2020 ▸). The final mechanical properties result from the formation of twin grain boundaries (TWIP effect) within stable austenite grains and/or from the deformation-induced formation of martensite in individual austenite grains (TRIP effect) (Fonstein, 2015 ▸; Callister & Rethwisch, 2008 ▸). These steels exhibit high tensile strengths in the formed state with a large remaining deformation reserve.

Welding is the dominant joining process for steel components in automotive engineering, in particular with regard to the assembly of the car body (Kimchi & Phillips, 2023 ▸; Zuniga & Sheppard, 1997 ▸; Perret *et al.*, 2011 ▸). Although high-manganese steels are generally suitable for welding, they are affected by a number of challenges (Böllinghaus & Herold, 2005 ▸; Matteis *et al.*, 2014 ▸; Keehan *et al.*, 2010 ▸; Baptista *et al.*, 2018 ▸):

First, a high carbon content promotes the formation of coarse grains and also leads to the precipitation of carbides at low cooling rates.

Second, both primary austenitic solidification and low thermal conductivity facilitate hot cracking in the weld seam area, for example in the form of intergranular solidification cracks.

Third, the outstanding properties are the result of complex heat treatment and subsequent cold forming. Thermal joining leads to local melting and, as a result, recrystallization, which can severely impair the local material condition. In combination with the possible formation of coarse grains (see first point), this can lead to a deterioration in the mechanical properties, such as the fatigue strength. However, these properties are intricately adjusted by deformation in the first place, facilitating local plastic deformation during further processing or external strain.

When using high-manganese steels in the automotive sector, the interaction between welded joints and the formed base material has not yet been sufficiently investigated. In particular, the generation of residual stresses in the joint area can lead to local component failure. Failure to consider the superposition of macro- and microscopic residual stresses can lead to uncertainties in component design. In the present case, such residual stresses are introduced either by the welding process in the vicinity of the weld seam or by the previous sheet metal forming. In contrast, high tensile residual stresses are generally assumed in design practice, although neither the actual stress state nor the material properties, nor the resulting limits of the load-bearing capacity nor the ultimately permissible residual stresses, have been clarified conclusively (Hensel *et al.*, 2016 ▸). However, such conservative component design does not necessarily contribute to a more reliable design and is, at the same time, in direct contrast to the pursuit of efficiency through lightweight construction.

Knowledge of the material properties in the strain-hardened deformed state is required to be able to assess accurately the residual stresses near the weld seam. Therefore, this article addresses the deformation behavior of different steel grades, as components in the automotive sector are usually used in a formed state and subsequently welded. The steels examined are the purely austenitic TWIP steel X40MnCrAlV-19-2.5-2 and the TRIP steel HCT690T, with the common mild steel S355MC used as a reference. These materials were selected to investigate technically relevant alloys in automotive engineering while enabling the TWIP and TRIP mechanisms to be investigated separately. The deformation behavior after welding is critical and significantly influences passenger safety. After all, the integrity of the individual welding spots is of key importance in the event of a crash, as it affects the ability of the car body structure to absorb kinetic energy (De Cooman *et al.*, 2011 ▸; Zuniga & Sheppard, 1997 ▸). The rolling of the steel sheets, applied to the semi-finished product in a prior stage of the manufacturing chain, already leads to a directional dependence of the macroscopic mechanical properties in the as-delivered state, as a crystallographic texture or rather a preferred orientation of the crystallites is formed as a result of the rolling process.

In order to investigate systematically the influence of the initial texture on the deformation behavior and the development of the texture due to material deformation, samples were subjected to tensile deformation using quasi-static testing. Quantitative phase analyses and analyses of the crystallographic texture indicated changes in both the microstructure and the anisotropy of material properties resulting from uniaxial deformation. From an application perspective, the effect of these changes on lattice strain distributions is of particular interest as it directly affects how readily the residual stresses can be evaluated. In this regard, a focus was placed on the oscillating distributions of *d*^{*hkl*}^ versus 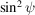, which were determined using Cr *K*α radiation.

The knowledge gained about the deformation-induced material anisotropy was then applied to the results of X-ray diffraction (XRD) experiments on a pre-deformed and welded sheet of steel HCT690T, which serves as an application example to illustrate the impact of material deformation. In particular, we investigated whether crystallographic texture or intergranular strain, caused by plastic deformation, has a stronger impact on the oscillating distributions of *d*^{*hkl*}^ versus 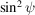 of the welded sample. Efforts were also made to consider the microstructural transition within the heat-affected zone using transformation calculations and weighting factors. Lastly, the impact of material anisotropy on how readily the residual stresses can be evaluated was investigated, depending on the present microstructure, the different influences on the oscillating distributions of *d*^{*hkl*}^ versus 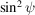 and the available ψ range of the investigation.

## 2. Materials and methods

### 2.1. Materials

The deformation behavior was analyzed for the TWIP steel X40MnCrAlV-19-2.5-2 (X40) and the TRIP steel HCT690T (also known as steel TRIP700, and therefore abbreviated herein as steel T7), with the mild steel S355MC (S3) as reference. Their respective designations according to DIN EN 10027-2 (Deutsches Institut für Normung, 2015 ▸) are 1.7401 (X40), 1.0947 (T7) and 1.0976 (S3).

The alloys were supplied as metal sheets with a thickness of approximately 2 mm. The T7 metal sheets were delivered in a galvanized state. Therefore, the Zn layer on the tensile specimens was removed before proceeding, using a 20% caustic soda solution at approximately 50°C and copper wire as catalyst. The removal of the Zn layer resulted in a final sheet thickness of about 1.75 mm in the case of the T7 sheets. The chemical composition of the three alloys was determined by spark optical emission spectroscopy and the results are listed in Table 1 ▸. Steel X40 is a purely austenitic steel. Steel T7 has an austenite content of approximately 17 vol.% in its delivery state (Brauser *et al.*, 2010 ▸), which leads to its high deformation reserve. The common mild steel S3 has a ferritic–pearlitic microstructure.

### 2.2. Tensile deformation

For the analysis of the deformation behavior and material anisotropy, dog-bone-shaped tensile samples following DIN 50125 (Deutsches Institut für Normung, 2022 ▸) of each alloy were cut from the metal sheets using water jet cutting.

Tensile tests were performed with a Zmart.Pro testing machine from Zwick Roell (Ulm, Germany) using a 200 kN load cell and mechanical wedge grips of type 8405L to clamp the samples. A Zwick Roell makroXtense mechanical sensor arm extensometer was utilized to enable high-resolution strain measurements. A measurement length of 60 mm was chosen.

A preload of 50 N was applied for testing and the samples were deformed using a displacement rate of 2.5 × 10^−3^ mm s^−1^, leading to an overall strain rate of approximately 4.2 × 10^−4^ s^−1^. A low strain rate was deliberately chosen to avoid a significant increase in the specimen temperature resulting from substantial plastic work, especially in the case of the highly formable steel X40.

For each alloy, the specimens were deformed to specific total strains. These total strain states, the stress at the maximum deformation and the plastic strain measured after unloading are listed in Table 2 ▸. In general, the samples were deformed in steps of about 5% of total strain, with the exception of a 2% step at the very beginning of the deformation. To avoid inhomogeneous deformation resulting from necking of the uniaxially deformed specimens, the maximum total strain was set lower than the uniform elongation of each steel. Hence, the maximum total strains applied are 45%, 25% and 20% for steels X40, T7 and S3, respectively.

### 2.3. Material isotropy and anisotropy

**Figure 1:**
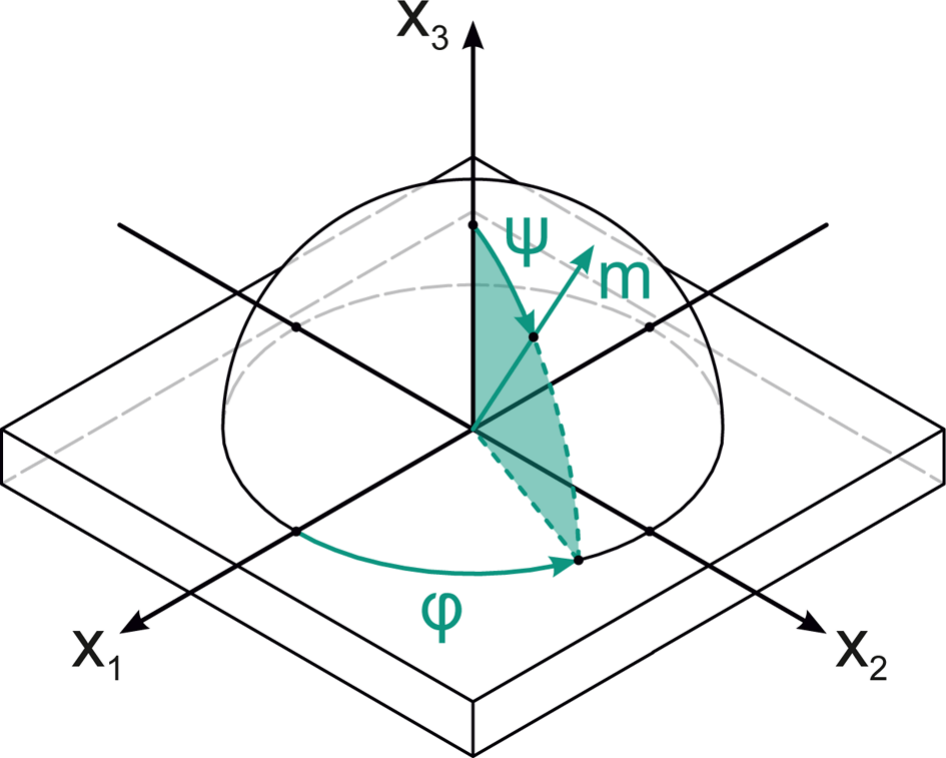
Definition of the orientation of the scattering vector **m** within the sample coordinate system using the azimuthal angle φ and polar angle ψ.

Two deformation-induced phase-specific characteristics are of particular interest in investigating the interaction between sheet steel and weld seam: the anisotropy of the material properties and the inhomogeneous microscopic residual stresses. In this context, Welzel and co-workers differentiate intrinsic elastic isotropy, macroscopic elastic isotropy (quasi-isotropy) and macroscopic elastic anisotropy (Welzel & Mittemeijer, 2003 ▸; Welzel *et al.*, 2005 ▸). Intrinsically elastic isotropy is expected for a polycrystalline material based on elastically isotropic crystallites. This is true for sophisticated mechanical metamaterials, for example, truss lattices or plate lattice structures (Tancogne-Dejean, 2018 ▸; Latture *et al.*, 2018 ▸; Chen *et al.*, 2024 ▸). In contrast, quasi-isotropic behavior may exist for materials consisting of elastically anisotropic crystallites. However, this requires the absence of both crystallographic texture and direction-dependent grain interactions to achieve macroscopically elastic isotropy (Welzel & Mittemeijer, 2003 ▸). High-strength steels, consisting of a body-centered cubic (b.c.c.) ferrite phase and/or a face-centered cubic (f.c.c.) austenite phase, can possess quasi-isotropic properties. In this case, their respective lattice strains ɛ and the associated stresses can be described in accordance with Hooke’s law, using the X-ray elastic constants (XECs) *s*_1_ and 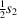 (Welzel & Mittemeijer, 2003 ▸; Evenschor & Hauk, 1975 ▸). In the case of laboratory XRD experiments in reflection geometry, the lattice strain distribution versus 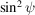 of an examined lattice plane set of type {*hkl*} can be simplified by assuming a planar stress state. This results in the following equation (Hauk, 1997 ▸): 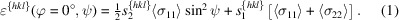
The determined lattice strain ɛ depends on the investigated lattice plane set of type {*hkl*}. The lattice strain ɛ is defined as a function of both the azimuthal angle φ and the polar angle ψ of the sample coordinate system. Both angles define the orientation of the scattering vector **m** within the sample coordinate system, as illustrated in Fig. 1 ▸. In this specific case, the azimuthal angle φ = 0° describes the direction of lattice strains investigated by the XRD experiment, with the corresponding principal stresses σ_11_ and σ_22_. The dependence of the polar angle is specified by 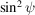.

In contrast to macroscopic elastic isotropy, macroscopic elastic anisotropy is the result of a pronounced crystallographic texture and/or direction-dependent grain inter­action. In industrial applications, high-strength steels have often undergone some degree of deformation. This deformation, whether caused by rolling, deep drawing or uniaxial deformation, leads to a change in the crystallographic texture. Plastic deformation induces direction-dependent intergranular strains. Elastic–plastic deformation results in a microscopically heterogeneous deformation of the individual crystallites of the polycrystals. For this reason, the elastic and plastic components of the deformation of differently oriented crystallites can differ. The different elastic strains lead to the formation of intergranular strains during unloading. The curve of the distribution of intergranular strain versus 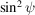 depends on the type of deformation (Hauk, 1997 ▸). The formation of intergranular strains does not require the presence of a pronounced crystallographic texture. However, crystallographic texture and plastic deformation influence each other, which is why their directional relationship also affects the formation of intergranular strains. Such strains affect the intergranular microstresses, which are examined by XRD experiments, and therefore can influence the analyses of phase-specific residual stresses. The effects accompanying plastic deformation affect TWIP and TRIP steels in particular, which have large plastic regimes and attain superior mechanical properties through intentional strain hardening. In the case of macroscopic elastic anisotropy, lattice strain determination is characterized by the following equation (Baczmanski *et al.*, 1995 ▸; Behnken, 2000 ▸): 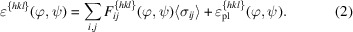
The anisotropic stress factors 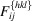 represent the sensitivity of the lattice strains to the mean stress tensor 〈σ_*ij*_〉. They can be determined using single-crystal elastic coefficients and the orientation distribution function (ODF) obtained by texture analysis (Behnken, 2000 ▸). Intergranular strains are accounted for using the term ɛ_pl_, which does not describe a plastic strain but represents the direction-dependent elastic intergranular strains resulting from plastic deformation of the polycrystalline material. As such, intergranular strains are often designated as part of the macroscopic elastic anisotropy, for example by Welzel & Mittemeijer (2003 ▸). As the present paper quantifies the two phenomena separately, the impacts on the lattice strain distribution generated by crystallographic texture and intergranular strains are referred to as elastic anisotropy and plastic anisotropy, respectively, in the following.

Knowledge of the superposition of both anisotropy effects is important as it can significantly influence how readily the residual stresses can be evaluated, including the area around the weld seam. In order to interpret the residual stresses in the weld seam area adequately, it is therefore essential to analyze the anisotropy effects, especially in the case of heavily deformed TWIP and TRIP steels.

### 2.4. X-ray diffraction analyses

Most of the XRD measurements presented in this publication were performed after the removal of 100 µm of near-surface material. The removal guaranteed that contamination, *e.g.* oxides and residues, and other surface effects resulting from preliminary preparation steps did not negatively influence the XRD investigations. The surface layers were electro­chemically removed using a LectroPol-5 in combination with A2 electrolyte, both provided by Struers ApS (Ballerup, Denmark). To avoid shadowing and edge artifacts during XRD analyses, an area of approximately 10 × 10 mm was removed. An exception to this procedure is the examination of the welded T7 sheet, for which the direct Zn-stripped surface was analyzed.

Residual stress analyses were performed using the 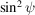 method (Macherauch & Müller, 1961 ▸). A three-circle diffractometer, operated in ψ mode and equipped with V-filtered Cr *K*α radiation (wavelength 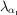
 = 2.29 Å), was applied. The primary aperture was a pinhole collimator with a nominal diameter of 1 mm. To measure the {220} X-ray interference line of the austenite phase, a 2θ = 0.4° slit aperture was placed in front of the scintillation counter. A 0.8° symmetrizing slit according to Wolfstieg (1976 ▸) was applied for the analysis of the {211} X-ray interference line of the ferrite phase. A total of 21 sample tilts, distributed equi­distantly versus 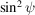, were considered within a ψ range of −60° ≤ ψ ≤ 60°. The high number of ψ tilts and the chosen ψ range are necessary to be able to demonstrate the expected nonlinearity of the 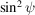 plot resulting from the deformation of the samples.

The X-ray interference lines were fitted using Pearson VII functions after linear background subtraction. The *K*α doublet of austenite was approximated using a double peak fit. The use of Cr *K*α radiation in XRD analyses of steels is well established in industry, mainly due to excellent measurement statistics and compatibility with both ferrite and austenite phases. For this reason, the α{211} and γ{220} X-ray interference lines of the ferrite and austenite phases are commonly investigated in laboratory X-ray diffraction experiments. However, the X-ray interference lines of type {*hh*0} in particular are more susceptible to anisotropy effects compared with some other X-ray interference lines. The lattice plane family γ{311}, which is much more robust against anisotropy effects, can be investigated using either Mn *K*α radiation, Co *K*α radiation or monochromated Cu *K*α radiation (Hutchings *et al.*, 2005 ▸). As part of the presented research project, several analyses based on the examination of the γ{311} X-ray interference line were performed using Co *K*α radiation. However, the possible high degrees of deformation in the case of steel X40 still lead to noticeable anisotropy effects.

In combination with the above-mentioned advantages of experiments using Cr *K*α radiation, the investigation of the γ{220} X-ray interference line appeared to be beneficial. To ensure comparability of the lattice strain distributions, the initially determined {*hkl*}-specific lattice plane spacings *d*^{*hkl*}^ of each phase are normalized as follows: 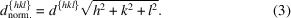
Phase-specific residual stresses were calculated using a weighted least-squares regression model in combination with the XECs and strain-free lattice parameters for the ferrite and austenite phases listed in Table 3 ▸. The XECs were determined by applying the Eshelby–Kröner model (Kröner, 1958 ▸) and the single-crystal elastic constants of the stiffness tensor provided by Every & McCurdy (1992 ▸) and Salmutter & Stangler (1960 ▸). The integral breadth (IB) values of the examined X-ray interference lines listed in this paper are the averages determined from all sample tilts with |ψ| < 30° to avoid a disproportionate effect of instrumental peak broadening when comparing different deformation states. In the case of the welded sample, the determined IB values were normalized using the IB value that was determined in the base material of the welded undeformed sample.

A series of quantitative phase analyses were performed to quantify the austenite phase fraction within the T7 samples. For this purpose, a θ–2θ diffractometer was applied using Zr-filtered Mo *K*α radiation (wavelength 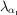
 = 0.71 Å). The primary aperture was a 1 × 1 mm slit collimator, and a 0.4° slit was used as the secondary aperture in front of the scintillation counter. A 2θ range from 20° to 58° was examined with a 2θ increment of 0.1°. Three independent separable X-ray interference lines of each of the ferrite and austenite phases are located within the measurement area and were utilized for the determination of the austenite phase fraction. These X-ray interference lines are {200}, {211} and {321} in the case of the ferrite phase and {200}, {220} and {311} in the case of the austenite phase. The phase fraction of austenite was calculated according to ASTM E975-22 (ASTM, 2023 ▸). The standard deviation of the results was estimated using a parameter sensitivity analysis.

The analyses of crystallographic texture were carried out using a four-circle diffractometer and Fe-filtered Co *K*α radiation (wavelength 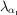 = 1.79 Å). A pinhole collimator with a nominal diameter of 1 mm was used as the primary aperture and a secondary slit aperture of 0.8° was placed in front of the scintillation counter. Incomplete pole figures were measured with a polar angle range of 0° ≤ ψ ≤ 70° and an azimuthal angle range of 0° ≤ φ ≤ 355°, each with a step size of 5°. In the case of the austenite phase of steel X40, incomplete pole figures were determined for the {220}, {311} and {222} lattice planes. The texture examination of steels T7 and S3 is based on incomplete pole figures for the {200}, {211} and {220} lattice planes of the ferrite/martensite phase.

In the case of steel X40, the austenite phase was examined, whereas in the cases of steels T7 and S3, the ferrite phase was analyzed. Although the austenite phase fraction of steel T7 is initially high enough for examination, it drops to phase fractions below 10 vol.% at total strains of 15% or higher. These low phase fractions lead to insufficient data quality for residual stress and texture analyses, particularly in the deformed state. For this reason, the following results for steel T7 are limited to the ferrite phase.

All considerations of crystallographic texture within this publication assume orthorhombic specimen symmetry (Kocks *et al.*, 2000 ▸). This assumption is valid since both the austenite phase and the ferrite phase have cubic lattice structures and the specimens were carefully aligned within the diffractometers.

**Figure 2:**
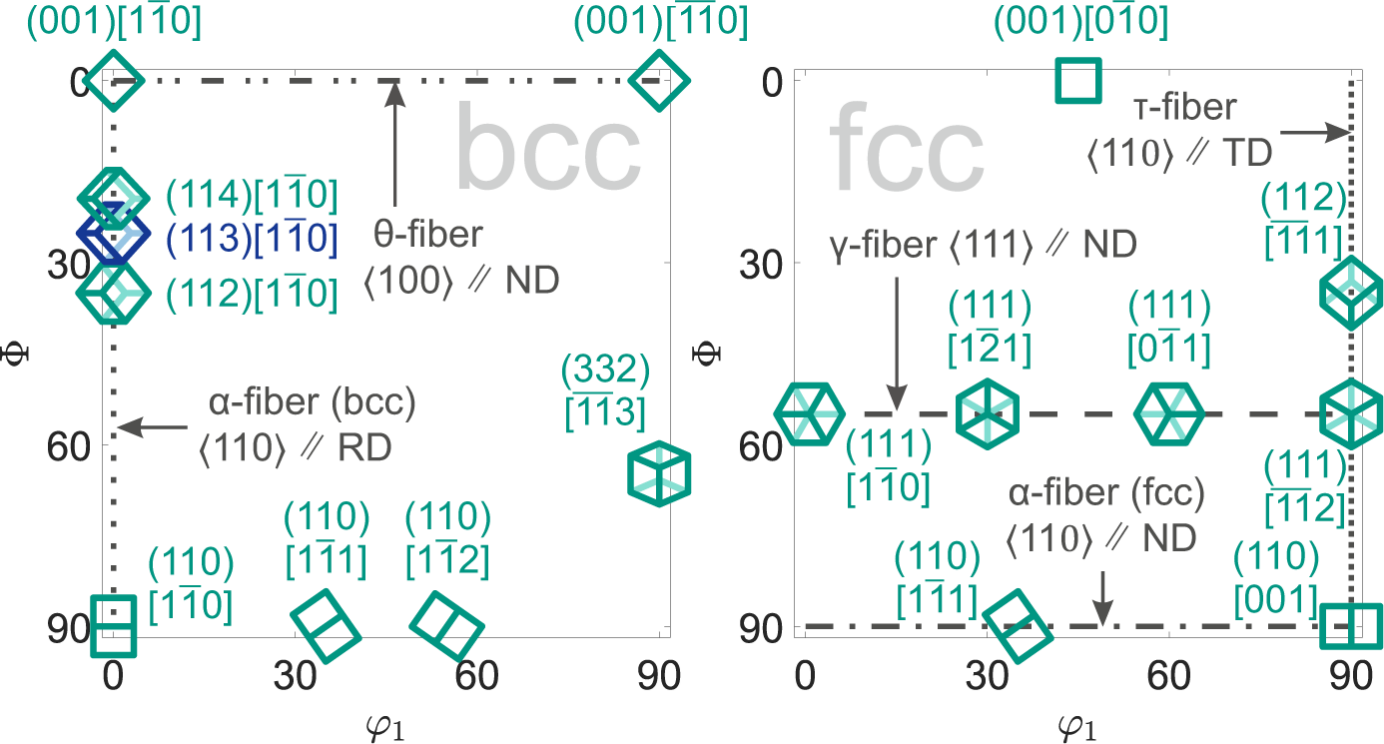
The φ_2_ = 45° cross section of the ODF in Bunge notation, showing important measured texture components of the b.c.c. lattice of the ferrite phase on the left and the f.c.c. lattice of the austenite phase on the right.

The open-source MATLAB *MTEX* toolbox (Bachmann *et al.*, 2010 ▸) was used to calculate the ODF, the texture components and their respective volume fractions. The calculation of the ODF is based on three incomplete pole figures, which are mentioned above, for each phase. Fig. 2 ▸ visualizes the important and observed texture components of the ferrite and austenite phases, and Table 4 ▸ provides the Bunge notation and Euler angles of the illustrated texture components.

Stress factors were determined using the *ISODEC* software (Gnäupel-Herold, 2012 ▸). To highlight the increasing non­linearity of the stress factors 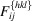 versus 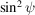 with increasing texture, the calculation of the elastic constants was performed for the grain interaction models of Voigt (1886 ▸), Eshelby–Kröner and Reuss (1929 ▸). Their calculation is based on the single-crystal elastic constants provided by Every & McCurdy (1992 ▸) and Salmutter & Stangler (1960 ▸) and the ODF determined using *MTEX*.

Both quasi-isotropic and anisotropic assumptions were applied to the measurement data. Using nonlinear least-squares fitting, the compatibility of the different models with the measurement data was determined using the adjusted coefficient of determination 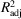. It is defined as follows (Rawlings *et al.*, 1998 ▸):
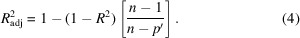
The parameter *n* represents the sample size of the present dataset and *p*′ is the number of model parameters. *R*^2^ is the conventional coefficient of determination resulting from the sum of the squared error (SSE) and the sum of the squared total (SST) (Kutner *et al.*, 2004 ▸):
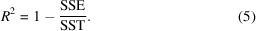
In contrast to the conventional *R*^2^, 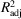 is corrected for the influence of the degrees of freedom. Therefore, it is well suited for the comparison of models with different numbers of parameters and is not subject to overfitting resulting from an increase in the complexity of the model (Rawlings *et al.*, 1998 ▸).

### 2.5. Welding

**Figure 3:**
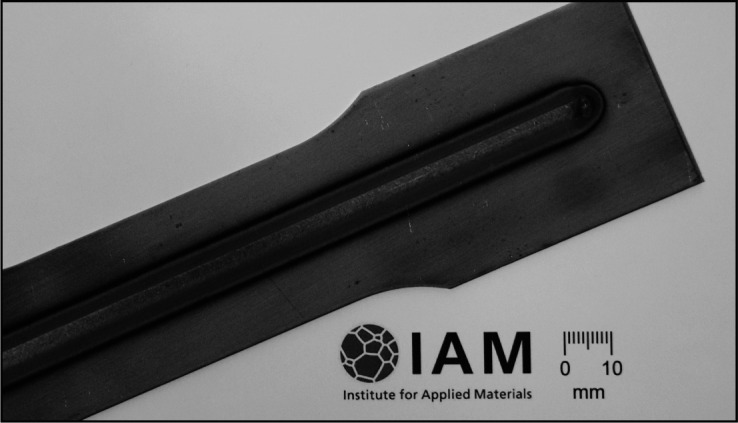
Illustration of a weld placed on a pre-deformed tensile sample of steel HCT690T.

A welded T7 sample is discussed as an example application. The sample is a flat tensile specimen with a total length of approximately 250 mm and a gauge width of approximately 40 mm, cut along the rolling direction from the metal sheet. It was pre-deformed to a total strain of 20% and afterwards tungsten inert gas bead-on-plate welded with a line energy of about 2.06 kJ cm^−1^. The chosen line energy results in a homogeneous weld with a width of approximately 6 mm, which passes evenly through the ∼1.75 mm thick metal sheet. The weld was placed in the middle of the sample along the primary deformation direction, as depicted in Fig. 3 ▸. The welds have a length of approximately 200 mm and run from one shoulder to the other of the tensile sample. During welding, the specimen was clamped to prevent weld distortions of the thin metal sheet due to thermal stresses. The residual stress analyses were performed in the middle section of the tensile specimen.

### 2.6. Metallographic analyses

The samples were prepared by grinding and polishing. For sequential grinding, SiC abrasive paper was used with progressively finer grit sizes, ranging from P120 to P2500. The polishing steps were performed using ethanol-based lubricant in combination with diamond suspension slurries (particle sizes of 6, 3 and 1 µm) and finally an aqueous solution with an oxide suspension slurry (particle size of 0.25 µm). Subsequently, the purely austenitic X40 samples were etched using the Beraha-II color etchant, and the mainly ferritic T7 and S3 samples were etched with the Beraha-I color etchant and a 2% Nital solution for grain boundary etching to visual­ize the microstructure (Beraha, 1966 ▸; Weck & Leistner, 2023 ▸). Metallographic examinations were performed using an Axiovert 200 inverse light optical microscope from Carl Zeiss AG (Oberkochen, Germany).

## 3. Results and discussion

### 3.1. Formation of a crystallographic deformation texture

Texture analyses were performed for samples in the as-delivered state as well as up to ten deformed states. The instrumental framework conditions are described in detail in Section 2.4. In addition to the ODFs provided here, the supporting information contains ODFs for further degrees of deformation and a complementary analysis of the present texture components.

Differences in the textures resulting from rolling and uniaxial deformation are due to the different active slip systems and stacking fault energies (SFEs) of the respective alloys. At room temperature, the dominant slip system for the austenite phase (steel X40) with an f.c.c. lattice is {111}〈110〉. The low SFE of the TWIP steel X40 and the associated capacity for deformation twinning enable the formation of additional crystal orientations (Randle, 1999 ▸; Vercammen *et al.*, 2004 ▸; Saleh *et al.*, 2013 ▸; Haase & Barrales-Mora, 2018 ▸). In contrast, the b.c.c. lattices of the ferrite phase (steels T7 and S3) exhibit, independent of temperature, a slip direction 〈111〉 with several deformation modes, such as {110}〈111〉 slip, {112}〈111〉 slip, {123}〈111〉 slip and {112}〈111〉 twinning (Rosenberg & Piehler, 1971 ▸; Jackson, 1991 ▸; Weinberger *et al.*, 2013 ▸).

**Figure 4:**
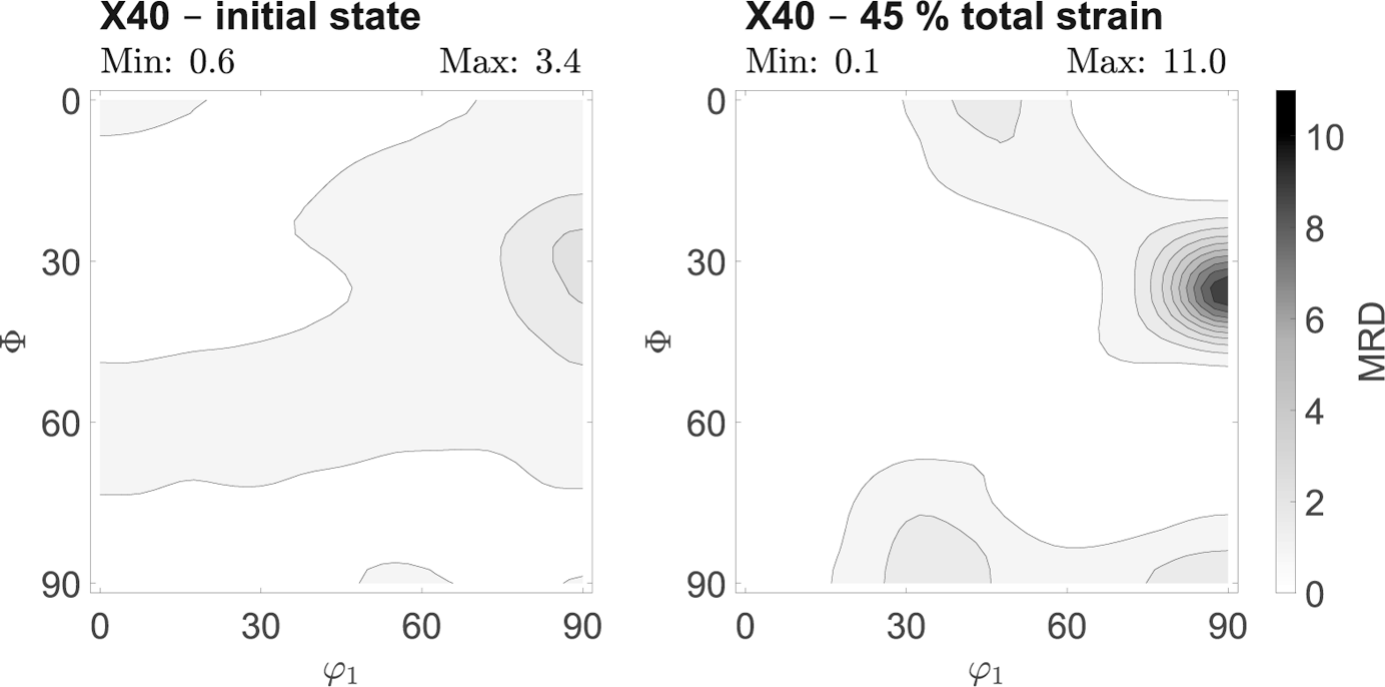
Texture evolution of steel X40MnCrAlV19-2.5-2 deformed in the rolling direction of the metal sheet. The cross section of the ODF at φ_2_ = 45° is illustrated for two degrees of deformation. The displayed levels are steps of 1.0 × multiples of random distribution.

The texture evolution for the austenitic steel X40 is illustrated by the ODFs depicted in Fig. 4 ▸. The initial state of steel X40 shows a γ-fiber {111} ∥ ND and the copper component {112}〈111〉. Next to a pronounced copper component in the deformed state, the Goss component {110}〈111〉, the cube component {001}〈010〉 and the A component {110}〈111〉 form as a result of plastic deformation. The ODF cross section for the initial state indicates a maximum multiples of random distribution (MRD) value of approximately 3.4, indicating a generally weak rolling texture. In contrast, a sheet deformed to a total strain of about 45% shows a strong deformation texture.

**Figure 5:**
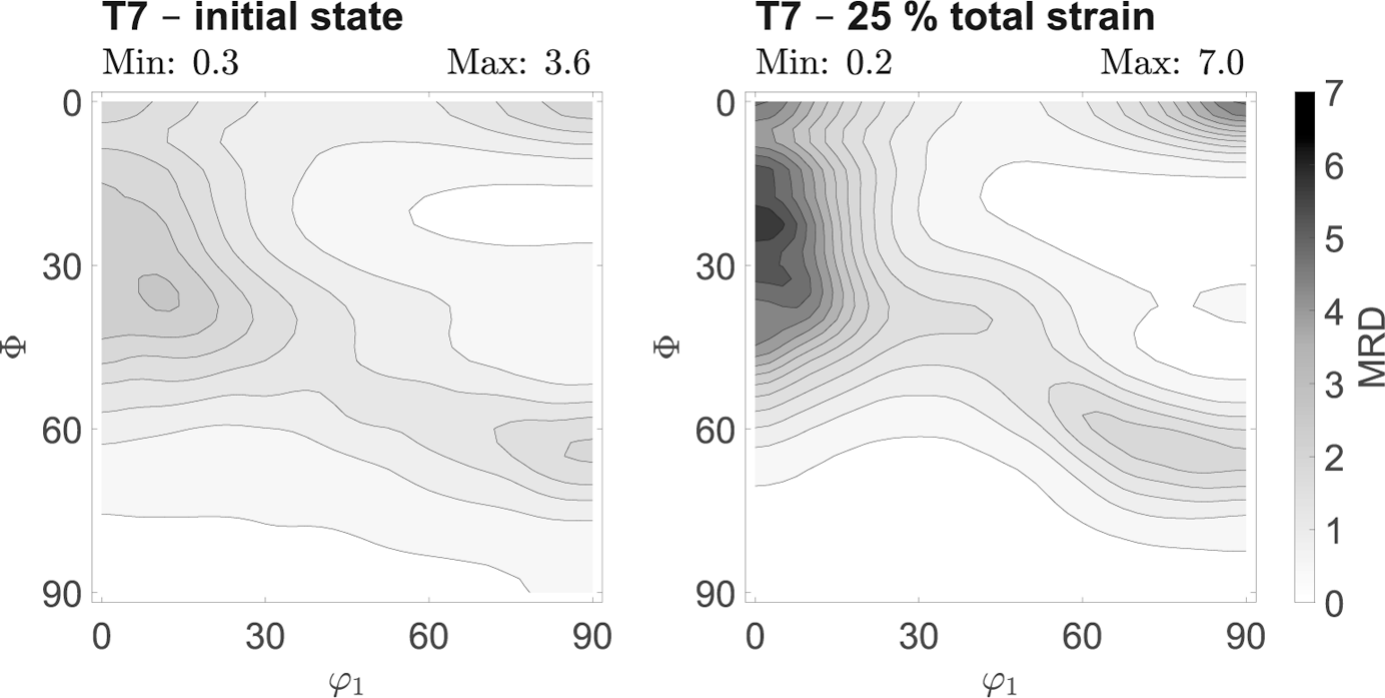
Texture evolution for the ferrite phase of HCT690T deformed in the rolling direction of the metal sheet. The cross section of the ODF at φ_2_ = 45° is illustrated for two degrees of deformation. The displayed levels are steps of 1.0 × multiples of random distribution.

In the case of TRIP steel T7, the crystallographic texture of the ferrite phase was examined. Two corresponding ODFs are shown in Fig. 5 ▸. The figure emphasizes that the crystallographic texture becomes more pronounced through uniaxial deformation, but the initial texture components remain present and gain in volume fraction and intensity. A total of five texture components can be derived, including the transformed copper components {112}〈110〉, {113}〈110〉 and {114}〈110〉, the rotated cube component {001}〈110〉, and the transformed brass component {332}〈113〉. The first four mentioned components are part of the α-fiber (b.c.c.). The transformed copper components have the highest volume fraction and MRD values. The initial state resembles the ODF determined in previous studies for rolled TRIP steels, which consists of the combination of rolling texture components of the ferrite phase and transformed rolling texture components of the austenite phase (Ray & Jonas, 1990 ▸; Hutchinson *et al.*, 1998 ▸; De Meyer *et al.*, 2001 ▸).

**Figure 6:**
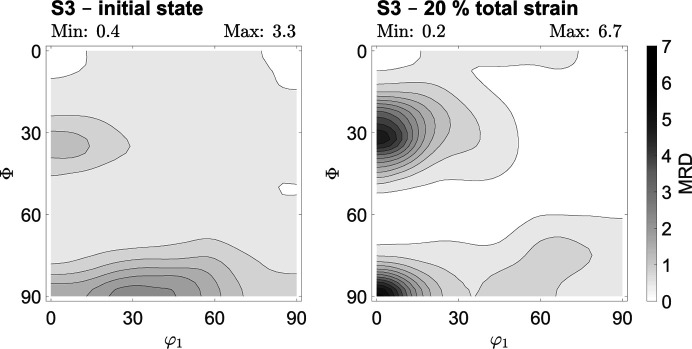
Texture evolution of steel S355MC deformed in the rolling direction of the metal sheet. The cross section of the ODF at φ_2_ = 45° is illustrated for two degrees of deformation. The displayed levels are steps of 0.5 × multiples of random distribution.

The ferritic–pearlitic mild steel S3 shows a different initial rolling texture from the ferrite phase of steel T7, sharing only the transformed copper component {112}〈110〉. This is shown for two ODFs in Fig. 6 ▸, and further data are given in the supporting information. Beyond that, the rotated Goss component {110}〈110〉, the A component {112}〈111〉 and the brass component {110}〈112〉 can be detected. As for steel T7, the initially present texture components are present throughout the uniaxial deformation. In contrast to TRIP steel T7, the mild steel S3 shows dynamic strain aging and a near-ideal elastic–plastic stress–strain curve, or rather minimal strain hardening (Knežević *et al.*, 2020 ▸; Prosgolitis *et al.*, 2021 ▸; Langlois *et al.*, 2022 ▸).

In all three examined materials, an overall low initial crystallographic texture resulting from the rolling process can be quantified. The initial maximum MRD values for the examined ODF cross sections are very similar for all three materials, ranging from about 3.3 to 3.6. During uniaxial deformation, a more distinct deformation texture evolves. For all three investigated steels, the strongest texture components created by the rolling process remain and their significance increases during further deformation in the rolling direction. The MRD values determined for the deformed state are high for all the steels examined here and remain similar when comparing the same degrees of deformation, for example 20% of total strain. In total, the MRD value increases on average by about 0.14 to 0.17 per percent of total strain, which can be seen in particular from the data in the supporting information. The final deformation texture of steel X40 is significantly stronger than that of steels T7 and S3 because the uniform elongation limit is much higher. In all cases, pronounced crystallographic textures are created as a result of the deformation process. As strong textures lead to macroscopically anisotropic material properties, it is necessary to examine whether the local analysis of residual stresses in these deformation states requires the prior determination of stress factors 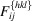 and whether it is sufficient to consider elastic anisotropy in this way.

### 3.2. Determination of stress factors

**Figure 7:**
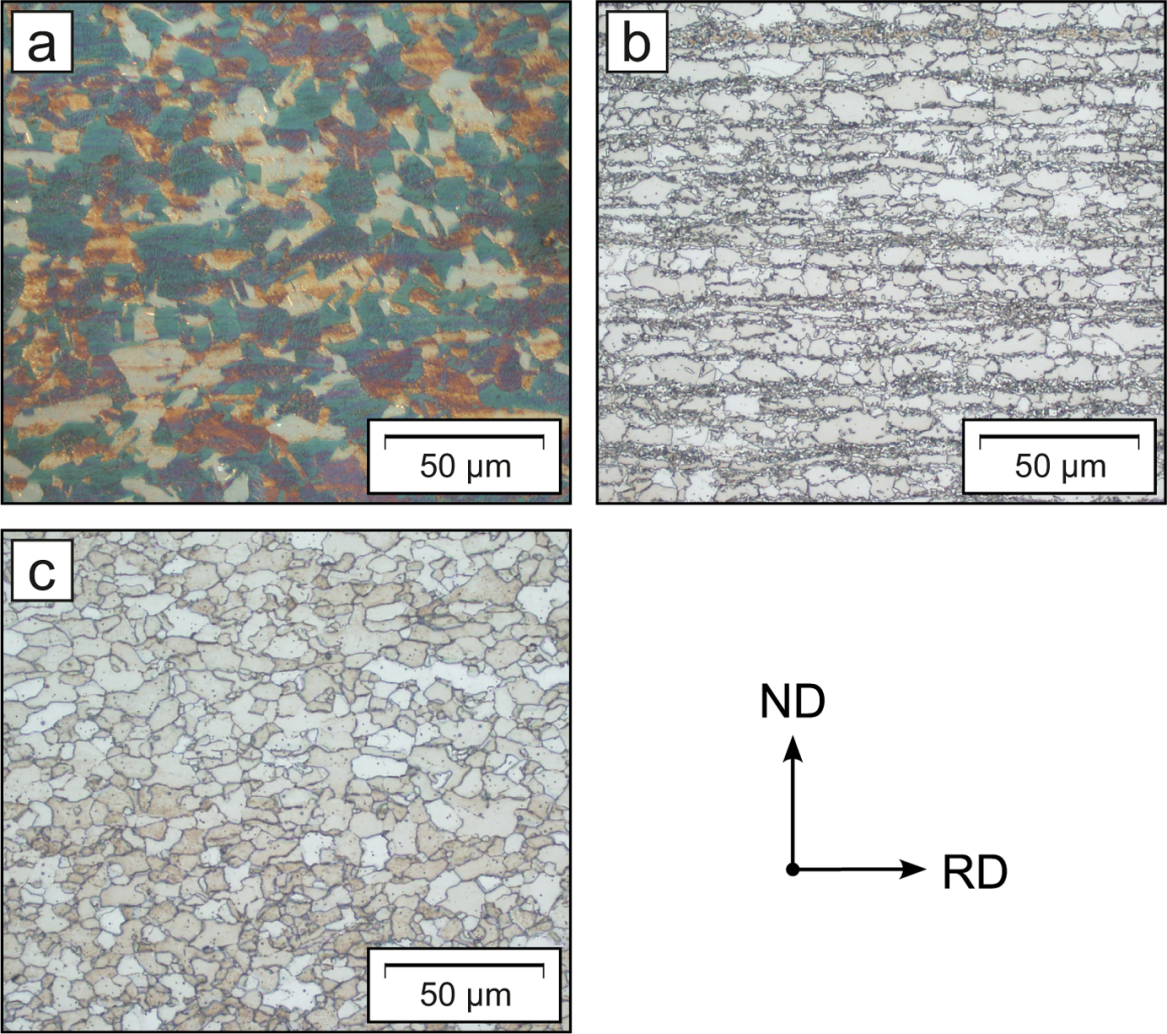
Optical microscope images taken of the examined steels. The sample coordinate system is given by the RD and ND. (*a*) The austentic TWIP steel X40MnCrAlV-19-2.5-2, using the Beraha-II etchant and a differential interference contrast. (*b*) The TRIP steel HCT690T etched with Beraha-I. (*c*) The ferritic steel S355MC etched with Beraha-I.

As significant elastic anisotropy must be assumed due to the evolution of strong deformation textures for the materials examined here, the stress factors 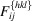 were calculated from the determined ODF. A uniform grain shape was assumed. Fig. 7 ▸ shows the microstructure of the investigated steels. The average grain sizes of the investigated phases were 12, 13 and 12 µm for steels X40, T7 and S3, respectively. Metallographic investigations showed that the lateral dimensions of grains in the as-delivered rolled state do not exhibit a significant aspect ratio.

Although the rolling direction is visible in the case of Fig. 7 ▸ for steel T7, this results from a row-shaped clustering of the austenite grains in the rolling direction rather than a non-uniform grain shape of either ferrite or austenite grains. Overall, the austenite phase is finely dispersed at the grain boundaries of the ferrite grains. Brauser *et al.* (2010 ▸) also made this observation. The retrieved results are consistent with the weak initial rolling texture described in the previous sub­section, and they emphasize the mitigation effect of recrystallization annealing on both texture and grain morphology. Uniaxially deformed samples were investigated by means of optical microscopy as well. Those investigations showed that the grain size and its aspect ratio are largely independent of the degree of uniaxial deformation of up to 45% of total strain in the present cases, and the globular grain shape is maintained. Therefore, spherical grain shapes were assumed for the calculation of the elastic constants.

**Figure 8:**
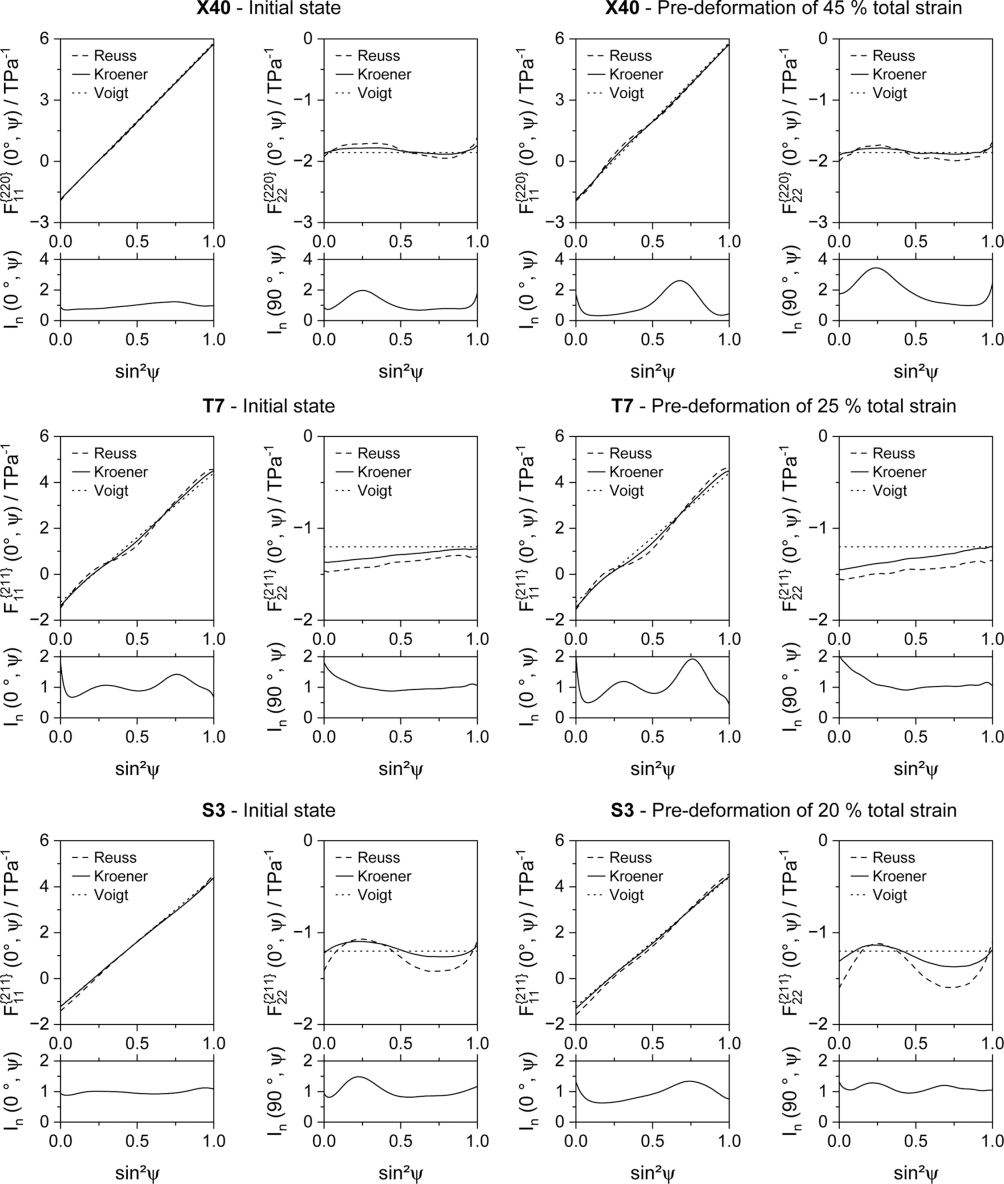
Stress factors and the ψ-dependent evolution of the normalized intensity *I*_n_, plotted versus 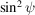. The parameters are given in longitudinal and transverse directions for the initial and highest deformed states of steels X40MnCrAlV-19-2.5-2, HCT690T and S355MC.

To preserve clarity, Fig. 8 ▸ only compares the stress factors 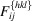 and normalized intensities for the initial and the most deformed state of each material. The stress factors 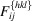 displayed were determined according to the grain interaction models of Reuss, Eshelby–Kröner and Voigt. In the following sections of this paper, only the stress factors 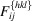 determined according to the Eshelby–Kröner model will be addressed, as the Reuss and Voigt models rather represent the physical boundary conditions, *i.e.* the lower and upper bounds. In addition to the stress factors 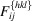, Fig. 8 ▸ plots the normalized intensities derived from the ODF against 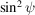 in the load direction (LD) and the TD for each state. Here, the normalized intensity constitutes an important indicator of the crystallographic texture, whereas the averaged intensity over the respective pole figure was used for normalization. As expected, the increasing texture leads to a more pronounced nonlinear distribution of 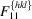 versus 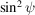. At first glance, the stress factors 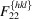 in the TD may appear to have a stronger nonlinearity versus 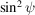. However, this is not true in absolute terms and is the result of the selected scaling of the *y* axis. When comparing the initial and deformed states, increasing nonlinearities in both stress factors and intensities versus 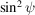 are recognizable. The texture evolution resulting from deformation is significant for all materials, as shown in Section 3.1, which is reflected by the strong increase in the oscillation of the intensity versus 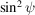 distributions.

### 3.3. Possibilities and limitations in the estimation of intergranular strains

**Figure 9:**
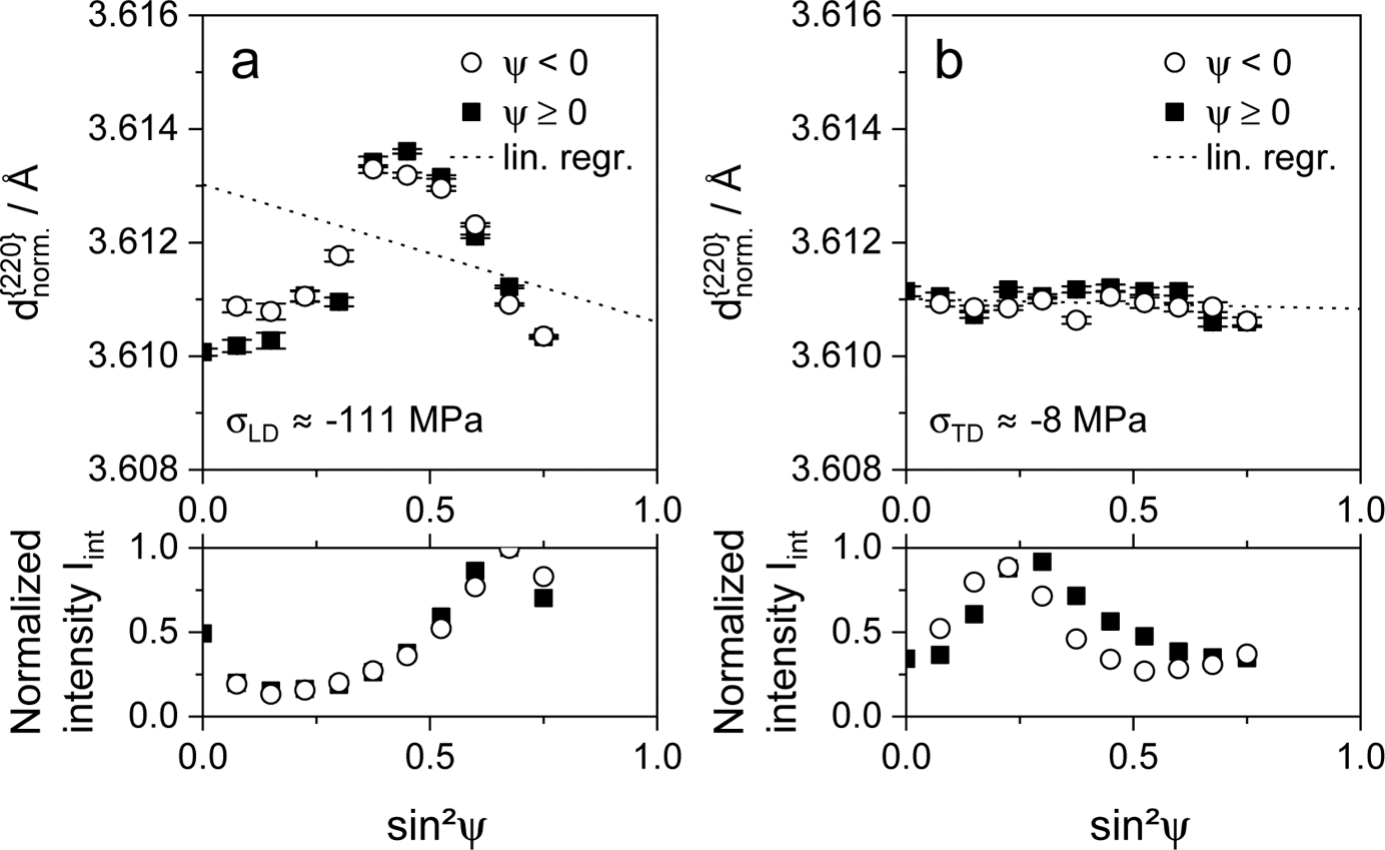
Distributions of lattice parameters 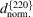 and normalized integral intensities *I*_int_ for the γ{220} X-ray interference line of TWIP steel X40MnCrAlV19-2.5-2, plotted versus 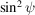. The specimen was deformed to a total strain of about 45% in the rolling direction of the metal sheet. The analyses were performed after unloading (*a*) in the load direction and (*b*) in the transverse direction.

**Figure 10:**
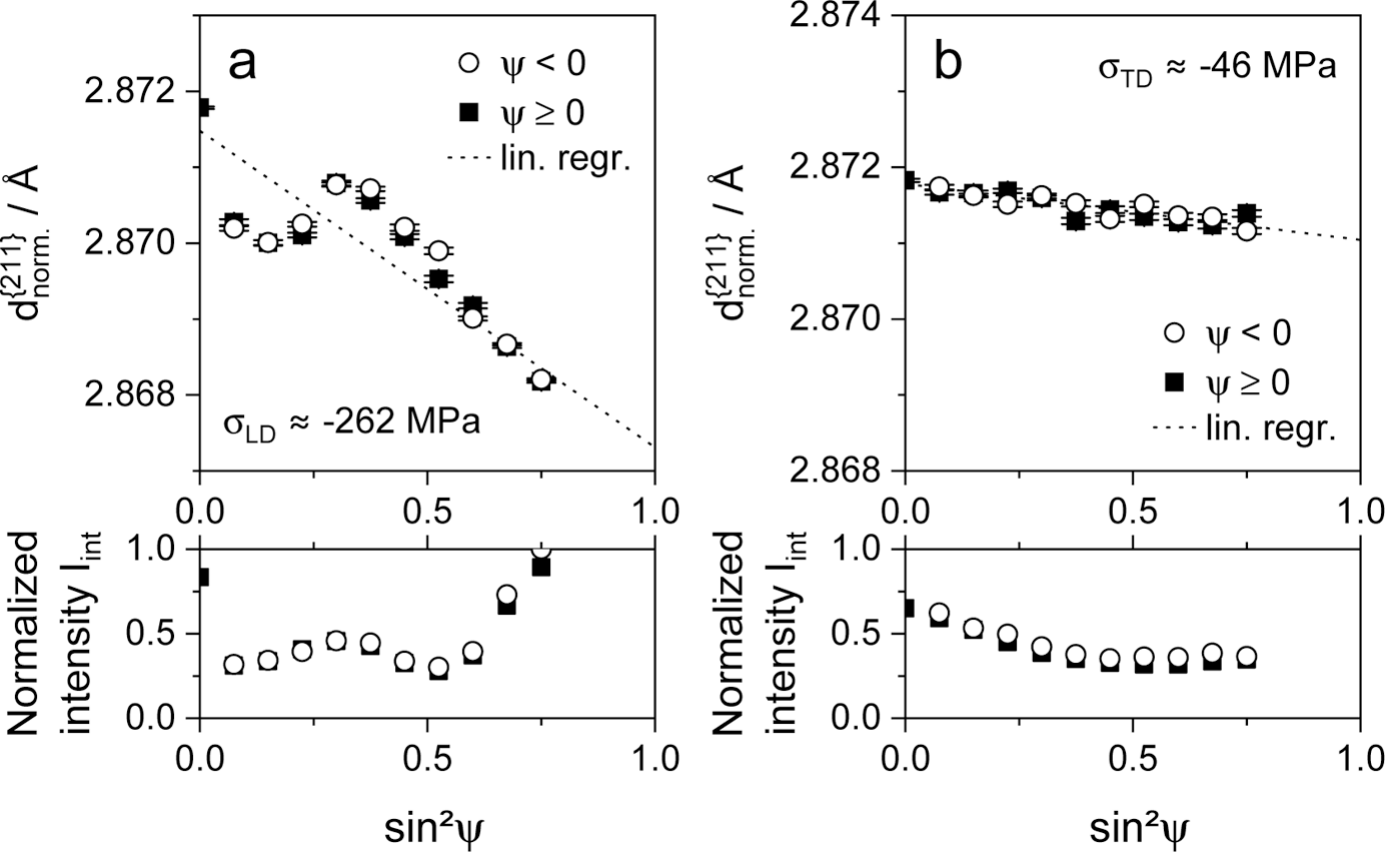
Distributions of lattice parameters 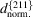 and normalized integral intensities *I*_int_ for the α{211} X-ray interference line of TRIP steel HCT690T, plotted versus 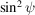. The specimen was deformed to a total strain of about 25% in the rolling direction of the metal sheet. The analyses were performed after unloading (*a*) in the load direction and (*b*) in the transverse direction.

**Figure 11:**
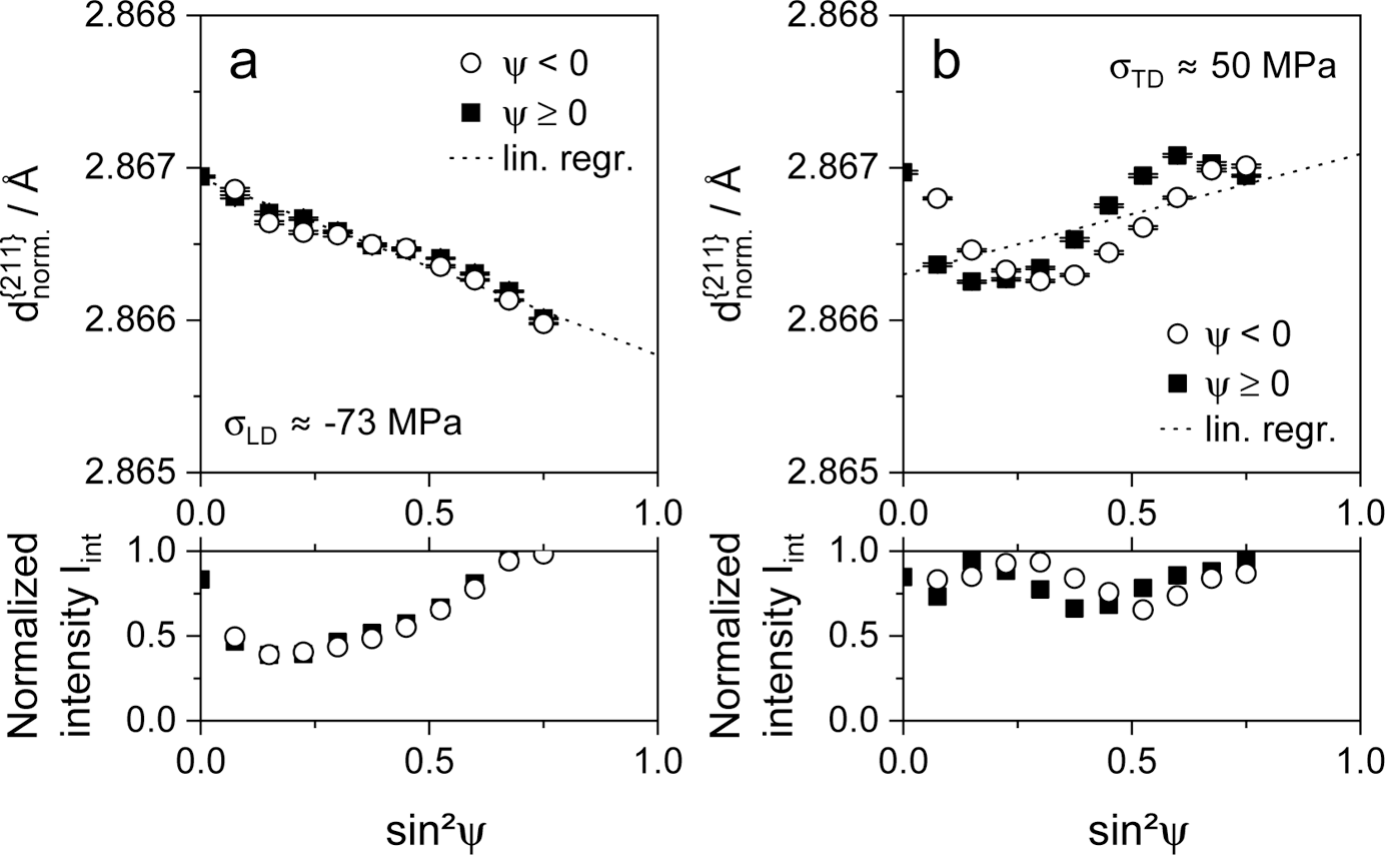
Distributions of lattice parameters 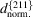 and normalized integral intensities *I*_int_ for the α{211} X-ray interference line of mild steel S355MC, plotted versus 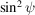. The specimen was deformed to a total strain of about 20% in the rolling direction of the metal sheet. The analyses were performed after unloading (*a*) in the load direction and (*b*) in the transverse direction.

In an attempt to quantify the plastic anisotropy effect, tensile specimens were deformed, unloaded and subsequently analyzed. The results of the residual stress measurements in the previous load and transverse directions are shown in Fig. 9 for steel X40, Fig. 10 for steel T7 and Fig. 11 for steel S3.

After the samples have been unloaded, no macrostresses are imposed. Therefore, the integral value of the microscopic residual stresses of the gauge volume should become zero in the case of the single-phase steel X40. How readily this expectation can be evaluated depends on the fact that both alloys are single phase and exhibit a sufficiently fine-grained microstructure. In this case, the gauge volume during laboratory XRD analyses contains an adequately large number of crystallites to average local microscopic residual stresses that are present because of a non-uniform distribution of plastic deformation on a granular level. Ideally, the superimposed strains from stresses and elastic and plastic anisotropy can be separated this way. As the first two mentioned components are stress related, only intergranular strains should remain in the stress-free unloaded case. Unloaded samples of steels T7 and S3 were also examined to assess the possibility of quantifying intergranular strains despite their respective two-phase microstructures.

In Fig. 9 ▸, the 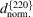 values and normalized intensities of the highest deformed state are plotted versus 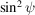 for an X40 sample. Fig. 9 ▸(*a*) depicts the results in the LD. The application of a conventional linear fit to calculate residual stresses, assuming quasi-isotropic behavior, determines compressive residual stresses of approximately −111 ± 66 MPa. The regression line of the linear fit is included as a dotted line, and it shows a poor correlation between the quasi-isotropic assumption and the displayed data. The value of the residual stresses determined this way does not appear to be reliable. The specified standard deviation of the residual stresses is derived from the goodness of fit of the regression line. Therefore, the deviation reflects the mathematical error and not the measurement uncertainty due to the present non­linearity. The measurement uncertainty can be expected to be significantly higher. For example, if a measurement range of |ψ| ≤ 45° (or rather 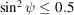) were considered, the conventional evaluation approach would lead to a residual stress value of about 374 ± 41 MPa due to the oscillating curve of the present 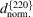 values versus 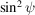. This result is well outside the 95% confidence interval of the investigation considering a range of |ψ| ≤ 60° (or rather 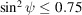), which accentuates the low reliability of the conventionally determined residual stress value in the present case.

Below the presentation of 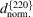 versus 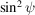, the normalized intensities are plotted versus 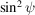. The highest measured intensity over 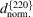 in both the LD and TD was used for normalization. However, due to good measurement statistics, it was possible to evaluate the {220} interference lines for all ψ tilts. The strong nonlinearity of the distribution of normalized intensities versus 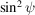 emphasizes the presence of a pronounced deformation texture resulting from a deformation to 45% of total strain, whereas there is good agreement with the results provided in Fig. 8 ▸. A comparably strong oscillation of the intensity versus 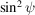 distribution was measured for the TD, as shown in part of Fig. 9 ▸(*b*). In contrast to those in the LD, the 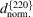 values in the TD feature a nearly linear curve versus 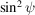. This allows the determination of more reliable residual stress values with significantly lower absolute deviations, for example approximately −8 ± 11 MPa in the present case. Due to the near-spherical grain morphology of the investigated phases, the high residual stress values determined in the LD are primarily the result of the nonlinearity, or rather intergranular strains, and no significant oscillating distribution of the 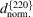 values versus 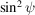, resulting from superimposed residual stresses, is apparently present in the LD. Otherwise, lateral contraction would effect distinct residual stresses in the TD. Therefore, the slope examined in the LD could be used for an approximate correction of the intergranular strains measured for the X40 samples, which were uniaxially deformed to about 45% of total strain.

The deformation of steel T7 leads to load partitioning between the ferrite and austenite phases and a resulting non-uniform deformation of the respective phases, which originates from the differences in the elastic and elasto-plastic properties of the two phases austenite and ferrite. A phase transformation of austenite to martensite is also caused by deformation. As a result, significant residual stresses form when the deformed sample is unloaded. In the example illustrated in Fig. 10 ▸(*a*), compressive residual stresses of approximately −262 ± 23 MPa are present within the ferrite phase. Although the nonlinearity of the 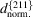 versus 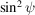 distribution is pronounced, the relative deviation is significantly smaller than in the case of steel X40. This is caused not only by the higher degree of deformation of steel X40 and the phase-related investigation of a different lattice plane family but also by the stress-related slope of the graph. There is a distinct intensity slope versus 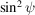 in the LD for steel T7. In comparison with those of the LD, the distributions of the 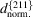 values (indicating residual stresses of about −46 ± 5 MPa) and of the intensities show less pronounced gradients versus 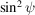 in the TD, as depicted in Fig. 10 ▸(*b*).

This approach is not applicable for the S3 sample, which was deformed to a total strain of approximately 20% in the RD of the metal sheet. Fig. 11 ▸(*a*) shows a clear gradient of the distribution of 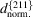 versus 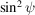 in the LD, which can be attributed to residual stresses. The conventional analysis, using a linear regression line based on the assumption of quasi-isotropic XECs, determines residual stresses of about −73 ± 3 MPa. The deviation is very low, partly because the plot shows only a marginal nonlinearity. However, the crystallographic texture in the LD is pronounced, as can be deduced from the slope of the normalized integral intensity versus 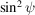. In the case of steel S3, a distinctly nonlinear distribution of 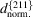 versus 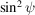 can be seen in the TD, as depicted in Fig. 11 ▸(*b*). A residual stress value of approximately 50 ± 15 MPa can be evaluated. As with steel X40, it can be assumed that the residual stress value determined is highly dependent on the investigated ψ range. At the same time, there is no significant intensity gradient versus 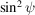. The phase fraction of pearlite is presumably low and therefore no significant load partitioning between ferrite and pearlite phases is expected. The lever rule (Callister & Rethwisch, 2008 ▸), taking a carbon content of approximately 0.022 ma.% into account, as noted in Table 1 ▸, gives a pearlite phase fraction lower than 2 vol.% in the case of thermodynamic equilibrium. However, steel S3 still exhibits residual stresses that remain after unloading. In addition to load partitioning between the ferrite and pearlite phases, residual stresses can remain after unloading within areas which are plastically deformed, creating Lüders bands. These stresses are induced in both the LD and TD (Hutanu *et al.*, 2005 ▸; Zhang & Kyriakides, 2022 ▸). The remaining stresses make it impossible to derive a substitute function for the intergranular strains from the unloaded tensile specimen, which could be used to correct the distribution of 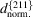 versus 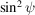 of a specific degree of deformation by the influence of these intergranular strains.

For all three alloys, substantial differences in the peak intensities versus 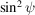 were detected in the LD and/or TD as a result of pronounced deformation textures. The intensity distributions and their respective orders of magnitude, determined by residual stress analyses, match the intensities recalculated from the pole figures, which are depicted in Fig. 8 ▸. This was anticipated, as the intensity values derived from residual stress analysis represent partial sections of the respective pole figures. Therefore, a superposition of both elastic and plastic anisotropy effects must be expected if local direction-dependent intergranular stresses are superimposed by a stress-related distribution of 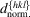 values versus 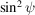, *e.g.* welding residual stresses in the case of the application example. However, this seems to be the case for all alloys. Steel X40 might be an exception, but the strong oscillation of the 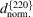 versus 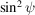 distribution, shown in Fig. 9 ▸(*a*), entails a non-negligible inaccuracy, which is caused by the insecure assumption of a stress-free state in the case of such a highly nonlinear distribution. Therefore, it does not seem possible to separate the superimposed elastic and plastic anisotropy effects on the basis of unloading of deformed samples. This behavior is comparable to the results obtained for the welded T7 sheet.

This means that the exact determination of the intergranular strain distribution requires unloading experiments, as they allow the targeted differentiation of two or more material states on the basis of 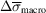. Especially in the case of two-phase materials, more than one unloading step from the load stage of interest is recommended, as stress reversal within the softer phase during unloading can lead to further local plastic deformation, which leads to a distortion of the quantifiable intergranular strains. The use of multiple unloading steps enables the comparison of states with large 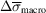 while preventing stress reversal beyond the yield strength of any phase. Using unloading steps after the previous loading, further evolution of the crystallographic texture can be ruled out. Therefore, the same stress factors 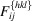 apply for different stress states, *i.e.* for the loaded and the entirely or partially unloaded state. However, the need to correlate macroscopic residual stresses and phase-specific residual stresses in the case of multiphase materials requires the determination of phase-specific stresses and the quantification of phase fractions (Hauk & Nikolin, 1988 ▸). This limits the characterizability of steel T7 by laboratory XRD experiments with respect to the determination of phase-specific residual stresses. Its inaccessibility is caused by the low fraction of the austenite phase, especially in the deformed state, and by the heterogeneous distribution of the austenite phase within the microstructure, as illustrated in Fig. 7 ▸(*b*). Therefore, other methods with larger gauge volumes and/or higher beam brilliance, such as high-energy synchrotron XRD experiments in transmission geometry, are better suited to characterizing two-phase steels with a low amount of the second phase, *i.e.* volume fractions lower than about 10% as for steel T7. At the same time, such investigations would enable the simultaneous acquisition of stress factors and plastic anisotropy for several interference lines. This approach is suitable for future work, as the results could also contribute to the validation of the laboratory results presented here.

### 3.4. Application example of a welded T7 steel sheet

**Figure 12:**
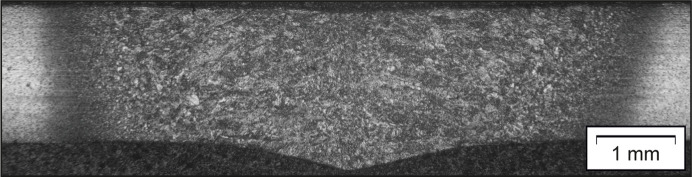
Optical microscope image taken of a T7 sheet, which was bead-on-plate welded with a line energy of 2.06 kJ cm^−1^, after Nital etching.

The knowledge gained with regard to the evolution of material anisotropy due to uniaxial deformation will now be transferred to an application example, a welded T7 metal sheet. It was uniaxially deformed to a total strain of approximately 20%, unloaded and subsequently bead-on-plate welded in the direction of tensile deformation. Therefore, the term LD is synonymous with the load, rolling, longitudinal and welding directions in the case of the welded T7 metal sheet. The strain of about 20% is well below the uniform strain of steel T7 at approximately 25% to avoid local necking and the associated inhomogeneities. Both deformation and welding were performed in the rolling direction of the specimen. The microstructure of a welded T7 sheet is depicted in Fig. 12 ▸.

**Figure 13:**
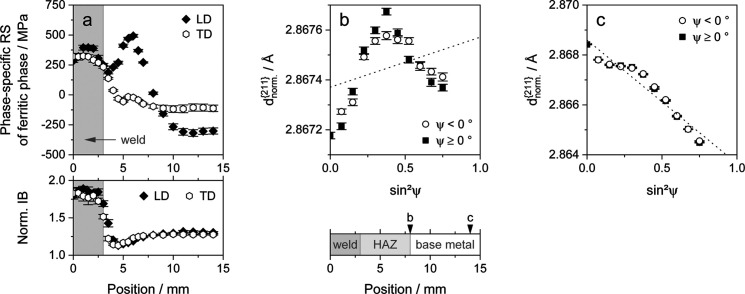
Phase-specific residual stresses (RS) created by welding of an HCT690T metal sheet, which was pre-deformed to a total strain of about 20% prior to bead-on-plate welding in the previous load direction. (*a*) The evolution of the phase-specific residual stresses of the ferrite phase depending on the distance to the weld seam center, determined in both the LD and the TD. Below the residual stresses, the associated evolution of the normalized mean integral breadth (IB_n_) of the X-ray interference lines is given. The 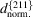 values determined in the LD are plotted versus 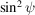 for distances of approximately (*b*) 8 mm and (*c*) 13 mm from the center of the weld seam.

Fig. 13 ▸ shows the results of residual stress analyses performed at the surface of the sample. The phase-specific residual stresses of the ferrite phase relative to the distance from the center of the weld are shown in Fig. 13 ▸(*a*). With a measurement range of approximately 14 mm on a path transverse to the weld line, the weld seam, the heat-affected zone (HAZ) and the surrounding base material were examined. Compressive stresses in the surrounding base material balance out the high tensile stresses in the weld seam and the HAZ. Due to the higher tensile stresses in the LD, the resulting compressive stresses in the base material are higher in this direction as well.

In general, the residual stress profile and magnitude of residual stresses determined match the results obtained by other studies of similar welded steel sheets (Ullner *et al.*, 2012 ▸; Reisgen *et al.*, 2011 ▸; Brauser *et al.*, 2012 ▸).

In addition to residual stresses, Fig. 13 ▸(*a*) contains the distribution of normalized integral breadth (IB_n_) values versus 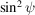, where the normalization was performed using the IB of the as-delivered state. The martensite/bainite formation within the weld and the associated high density of lattice defects result in rather high IB_n_ values (Macchi *et al.*, 2021 ▸; Ungár & Borbély, 1996 ▸). This discrepancy results from the degree of pre-deformation and the surface cleaning prior to welding. Considering the evolution of IB_n_, it can be assumed that the transition point from the HAZ to the base material is at a distance of about 8 mm from the weld center. Supplementary retained austenite analyses confirmed this transition point and determined an average retained austenite content of approximately 6.3 ± 2.0 vol.% within the base material.

Fig. 13 ▸(*b*) shows the investigated 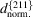 values versus 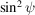, determined at a distance of approximately 8 mm from the weld center. At this approximate position, there is a zero crossing of phase-specific stresses, from tensile residual stresses in the seam and the HAZ to compressive stresses within the surrounding material. The nonlinearity of the distribution of 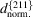 versus 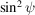 is pronounced. Fig. 13 ▸(*c*) depicts the distribution at a distance of about 14 mm from the weld center. High compressive residual stresses of approximately −300 MPa were determined at this position, leading to a clear gradient for the distribution of 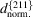 versus 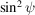. Nevertheless, the nonlinearity visible in Fig. 13 ▸(*b*) still applies to Fig. 13 ▸(*c*), but it has a lower relative weight due to the steep stress-related gradient of the 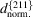 distribution versus 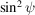.

Taking equation (2) into account, the high nonlinearity of the 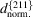 versus 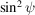 distribution close to the stress zero crossing, and the similar character of the nonlinearity in the case of the stressed base material, indicate the presence of intergranular strains. It can be assumed that they are superimposed by an elastic anisotropy effect due to the high welding residual stresses that can be found within the HAZ and the base material.

**Figure 14:**
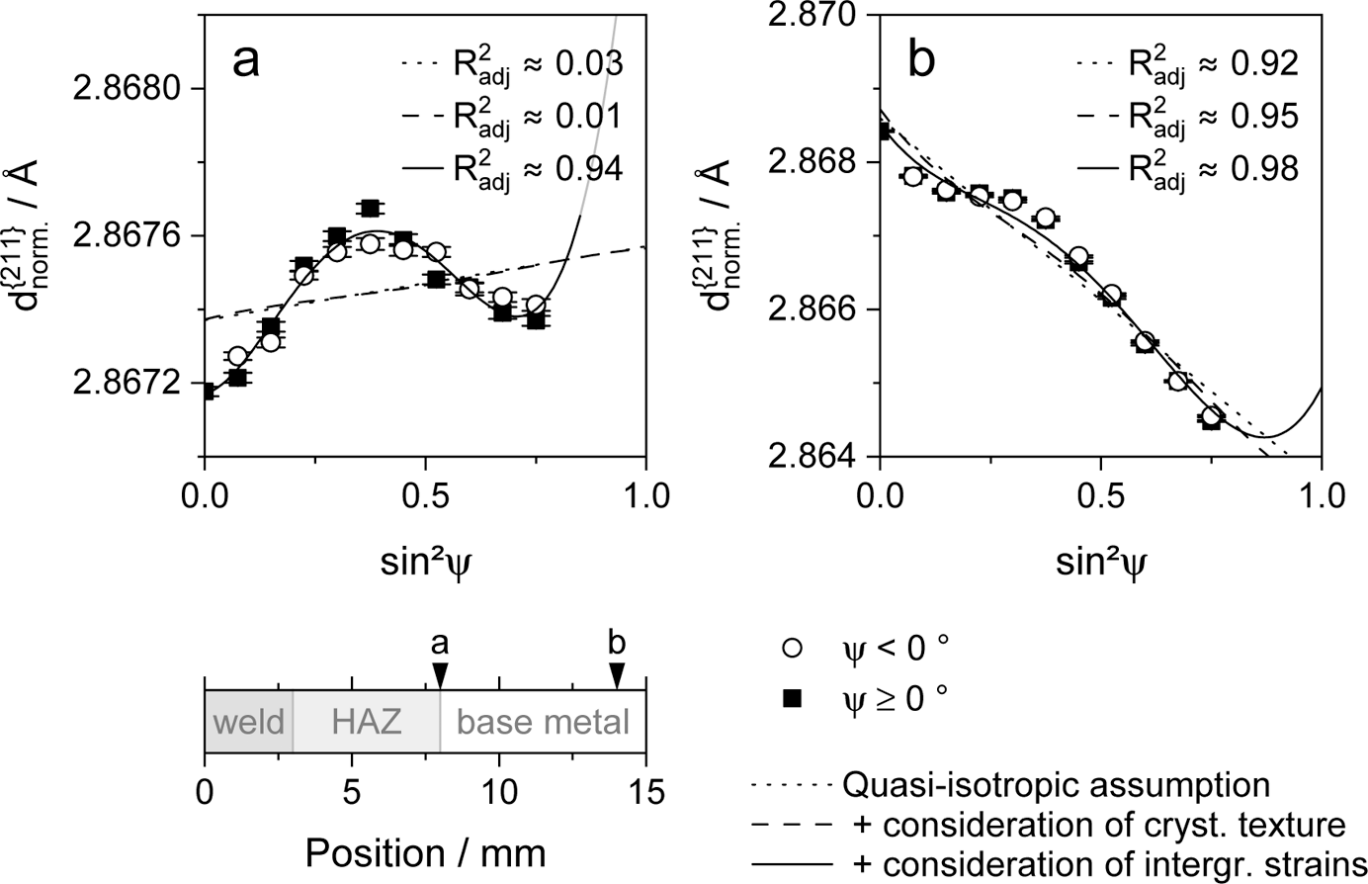
Nonlinear 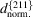 versus 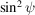 distributions determined for the welded HCT690T steel sheet in the longitudinal direction of the weld seam. The quasi-isotropic assumption is compared with methods considering elastic anisotropy and intergranular strains using the adjusted coefficient of determination 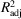. The evaluations are shown for distances of approximately (*a*) 8 mm, (*b*) 9 mm and (*c*) 14 mm from the center of the weld seam.

Applying the results of the material anisotropy investigations, the distributions of 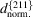 versus 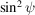 as shown in Fig. 14 ▸ can be determined. It compares the fitting accuracy of the conventional isotropic approach with the results obtained by assuming elastic and/or plastic anisotropy using the 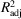 value. Both 〈σ_*ij*_〉 and *d*_0_ values were determined numerically using a least-squares approach. The graphs differ with respect to the present residual stresses, ranging from very low tensile residual stresses in Fig. 14 ▸(*a*) to rather high compressive stresses in Fig. 14 ▸(*b*). This comparison of the evaluations shows that the elastic anisotropy of the material alone does not suffice to describe the oscillating curve of 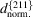 values versus 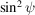. Comparing these two models, the 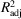 value is proportional to the present residual stresses.

If the nonlinear distribution of 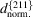 versus 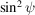 is not corrected by anisotropy effects, the facilitation of a large ψ range might help prevent a large misjudgment of residual stresses. However, laboratory measurements used in reflection geometry are mostly limited to a maximum polar angle of around |ψ| ≤ 70° due to the high absorption and broadening of the measurement spot at low beam incidence angles. Many laboratory setups, *e.g.* many mobile diffractometers dedicated to residual stress analysis, are even instrumentally restricted to |ψ| ≤ 45°. For the latter case in particular, the models for quasi-isotropy and elastic anisotropy are inadequate to describe the oscillating distribution of 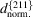 versus 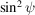, leading to an erroneous assessment of the present stresses. Correction for the plastic anisotropy effect seems to become essential. In order to maintain an approximate curve of intergranular strains versus 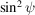, the stress gradient in the vicinity of the weld seam was utilized. It is assumed that the lattice strain distribution at the zero crossing at the reference position is mainly influenced by ɛ_pl_. The eventual low residual stresses are determined by a purely elastic approach and Δɛ results from plastic anisotropy. The available ψ range is expected to be large enough to obtain an equally distributed curve of ɛ_pl_ versus 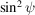. Therefore, the assumed residual stresses 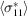 and 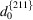 are numerically determined using the measured lattice strain distribution 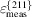 (φ = 0°, ψ) and applying equation (2), taking the elastic anisotropy into account based on the experimentally determined stress factors. The certainty of the acquired results depends on the deviation of the measuring spot from the zero crossing and the integral measurement of potentially thermally induced microstructure gradients resulting from primary beams with small collimation. In this way, the presented solution is sufficient in many cases but requires the analysis of a near-stress-free state. Alternatively, the intergranular strain distribution can be determined using unloading experiments.

However, superimposing the impacts on the 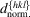 versus 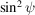 distribution generated by intergranular strains and stress factors 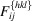 only enables the quantification of the 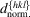 versus 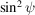 distribution within the base material. The heat input within the HAZ is suspected to result in a decreasing oscillation of the 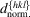 versus 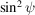 distribution, *e.g.* caused by recrystallization, recovery and grain growth.

## 4. Conclusions

The TWIP steel X40MnCrAlV-19-2.5-2 (1.7401), the TRIP steel HCT690T (1.0947) and, as a reference, the mild steel S355MC (1.0976) were characterized by laboratory X-ray diffraction stress and texture analyses. The focus of the investigations was on the characterization of the deformation behavior of the steels, more precisely the development of anisotropy effects depending on induced strain. For this purpose, samples were deformed to specific degrees of deformation using uniaxial tensile testing. Subsequently, the crystallographic texture and distributions of 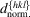 versus 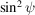 were analyzed to determine stress factors 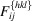 and estimates of intergranular strains ɛ_pl_, respectively. The results are important in better describing the interrelation between deformed steel and weld seams, which play a critical role in the application field of the mentioned materials. In addition, it seems possible to transfer the knowledge of the resulting material anisotropy to other technical issues, *e.g.* interactions with regard to sheet orientation and the resulting deformation behavior.

The following conclusions can be drawn from the investigations of the deformation behavior:

(i) As a result of different lattice structures, deformation mechanisms and reachable deformations, the textures generated vary for all investigated alloys. However, the crystallographic rolling texture, present in the initial state, is comparable for all investigated alloys, ranging between a maximum MRD of about 3.3 to 3.6. Depending on the highest degree of deformation, maximum MRD values between approximately 6.7 and 11.0 were reached after uniaxial deformation of the samples, whereas an average MRD increase of about 0.14 to 0.17 per percent of total strain could be identified.

(ii) On the basis of analyses of the crystallographic texture, an attempt was made to correct the nonlinearity of the 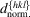 distribution using the stress factors derived from the orientation distribution functions. However, it could be shown that the stress factors are not sufficient to describe the obtained lattice strain distribution and only have a minor impact on the lattice strains. This applies to all alloys investigated and all degrees of deformation achieved. Instead, the intergranular strains resulting from plastic deformation seem to have the greatest influence on the lattice strain distribution. If they are disregarded, their relative impact on the determination of residual stresses is especially high in the case of low residual stresses.

As an application example, a welded HCT690T steel sheet was characterized and the findings regarding deformation-induced material anisotropy were applied. The changing microstructure within the heat-affected zone was also considered and corrective approaches were proposed.

The following conclusions can be drawn from the investigations of the interrelation between material anisotropy and the welded sample:

(iii) Typical tensile residual stresses can be identified within the weld seam and the heat-affected zone, which are balanced by compressive residual stresses in the base material. Using the evolution of the integral breadth of the X-ray interference lines and austenite phase fractions as a function of the distance to the weld center, the microstructural areas of weld seam, heat-affected zone and base material can be differentiated. The investigation of lattice strain distributions shows oscillating curves of 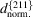 versus 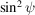, indicating a high degree of uncertainty in the evaluation of residual stresses, which strongly depends on the available ψ range. This again applies in particular to positions where low welding residual stresses are induced.

(iv) The distribution of intergranular strains versus 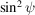 could be approximated using the zero crossing of residual stresses. However, it has been shown that the accurate quantification of these intergranular strain distributions necessitates unloading experiments, ideally with several unloading steps.

The results show that the high ductility of the steels examined here can result in significant material anisotropy. Knowledge of the extent of this anisotropy is essential for reliable characterization, especially of residual stresses.

## 5. Related literature

For further literature related to the supporting information, see Barbier *et al.* (2009 ▸), Bunge (1969 ▸), Chin *et al.* (1969 ▸), Chowdhury *et al.* (2005 ▸), Dillamore & Roberts (1964 ▸), Donadille *et al.* (1989 ▸), Haase *et al.* (2014 ▸), Jain *et al.* (2022 ▸), Lü *et al.* (2011 ▸), Nguyen-Minh *et al.* (2015 ▸), Paul *et al.* (2004 ▸), Saleh *et al.* (2014 ▸), Samek *et al.* (2008 ▸), Savoie *et al.* (1996 ▸), Tewary *et al.* (2014 ▸) and Wassermann & Grewen (1962 ▸).

## Supplementary Material

Additional data. DOI: 10.1107/S1600576725010350/xx5085sup1.pdf

## Figures and Tables

**Table 1:** Chemical compositions of the TWIP steel X40MnCrAlV-19-2.5-2, the TRIP steel HCT690T and the mild steel S355MC All alloying constituents are given in ma.%.

Alloy	C	Mn	Si	P	S	Al	V	Ti	Cr	Fe
X40	0.377	18.62	0.246	0.019	0.004	1.090	0.115	0.020	1.68	Balance
T7	0.176	1.793	0.366	0.011	0.006	1.279	0.008	0.004	0.02	Balance
S3	0.022	0.161	0.034	0.010	0.005	0.046	0.007	0.003	0.05	Balance

**Table 2:** Degrees of deformation of the investigated steels and the engineering stress at the respective strain states before unloading

Alloy	Total strain (%)	Plastic strain (%)	Engineering stress (MPa)
X40	2.0	1.7	455
	5.0	4.6	515
	10.0	9.5	601
	15.0	14.3	685
	20.0	19.2	751
	25.0	24.1	776
	30.0	29.0	806
	35.0	33.8	847
	40.0	38.8	855
	45.0	43.6	865
			
T7	2.0	1.7	568
	5.0	4.6	637
	10.0	9.5	692
	15.0	14.4	729
	20.0	19.4	750
	25.0	24.3	763
			
S3	2.0	1.8	431
	5.0	4.8	428
	10.0	9.7	450
	15.0	14.7	452
	20.0	19.6	447

**Table 3:** X-ray elastic constants 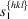 and 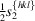 based on the single-crystal elastic constants provided by Every & McCurdy (1992 ▸) and Salmutter & Stangler (1960 ▸), and strain-free lattice parameter *a*_0_ (Wyckoff, 1963 ▸), used for the residual stress analysis

Phase	{*hkl*}	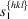 (TPa^−1^)	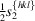 (TPa^−1^)	*a*_0_ (Å)
Ferrite	{211}	−1.233	5.700	2.8665
Austenite	{220}	−1.457	6.157	3.6469

**Table:** Most of these components appear on the φ_2_ = 45° cross section of the ODF. The fiber orientations are also provided, including their orientation depending on the rolling direction (RD), transverse direction (TD) and normal direction (ND) of the investigated samples. The rolled samples were uniaxially deformed in the RD by tensile testing.

Designation	Orientation {*hkl*}〈*uvw*〉	Euler angles (°) [φ_1_, Φ, φ_2_]	Fiber	Phase
Cube	{001}〈010〉	[45, 0, 45]	θ	f.c.c.
Rotated cube	{001}〈110〉	[0, 90, 45]	θ	b.c.c.
Goss	{110}〈001〉	[90, 90, 45]	α_f.c.c._, θ	f.c.c.
Rotated Goss	{110}〈110〉	[0, 90, 45]	α_b.c.c._, α_f.c.c._	b.c.c.
Copper	{112}〈111〉	[90, 35.3, 45]	β, τ	f.c.c.
Transformed copper	{112}〈110〉	[0, 35.3, 45]	α_b.c.c._	b.c.c.
Transformed copper	{113}〈110〉	[0, 25.2, 45]	α_b.c.c._	b.c.c.
Transformed copper	{114}〈110〉	[0, 19.5, 45]	α_b.c.c._	b.c.c.
Brass	{110}〈112〉	[54.7, 90, 45]	α_f.c.c._, β	b.c.c.
Transformed brass	{332}〈113〉	[90, 60.5, 45]	τ	b.c.c.
A	{110}〈111〉	[35.4, 90, 45]	α_f.c.c._	b.c.c., f.c.c.
E	{111}〈110〉	[0, 54.7, 45], [60, 54.7, 45]	γ	f.c.c.
F	{111}〈112〉	[30, 54.7, 45], [90, 54.7, 45]	γ	f.c.c.
S	{123}〈634〉	[59, 36.7, 63.4]	β	f.c.c.

**Table:** 

Fiber orientations	
α_b.c.c._	〈110〉 ∥ RD
α_f.c.c._	〈110〉 ∥ ND
β	〈110〉 tilted 60° from ND to RD
γ	〈111〉 ∥ ND
τ	〈110〉 ∥ TD
θ	〈100〉 ∥ ND

## Data Availability

The data presented in this study are available on request from the corresponding author.
