# Supplementary material for: Evolution of crystallographic texture and material anisotropy effects resulting from uniaxial deformation for high-strength steels with high manganese content
Source: J Appl Crystallogr. 2026 Feb 1;59(Pt 1):163–78. doi: 10.1107/S1600576725010350 (PMC12871490; doi:10.1107/S1600576725010350)
Supplement: Supplementary file 1 [file j-59-00163-sup1.pdf]

JOURNAL OF
APPLIED
CRYSTALLOGRAPHY

Volume 59 (2026)

Supporting information for article:

Evolution of crystallographic texture and material anisotropy effects resulting from uniaxial deformation for high-strength steels with high manganese content

Michael Zuern, Morteza Dadkhah, Thomas Nitschke-Pagel and Jens Gibmeier

1 Additional ODF data

1.1 X40MnCrAlV19-2.5-2 (X40)

Figure 15 depicts the ODFs determined for the austenitic TWIP steel X40. It was deformed up to a total strain of approx. 45 %, so as not to exceed the range of uniform elongation.

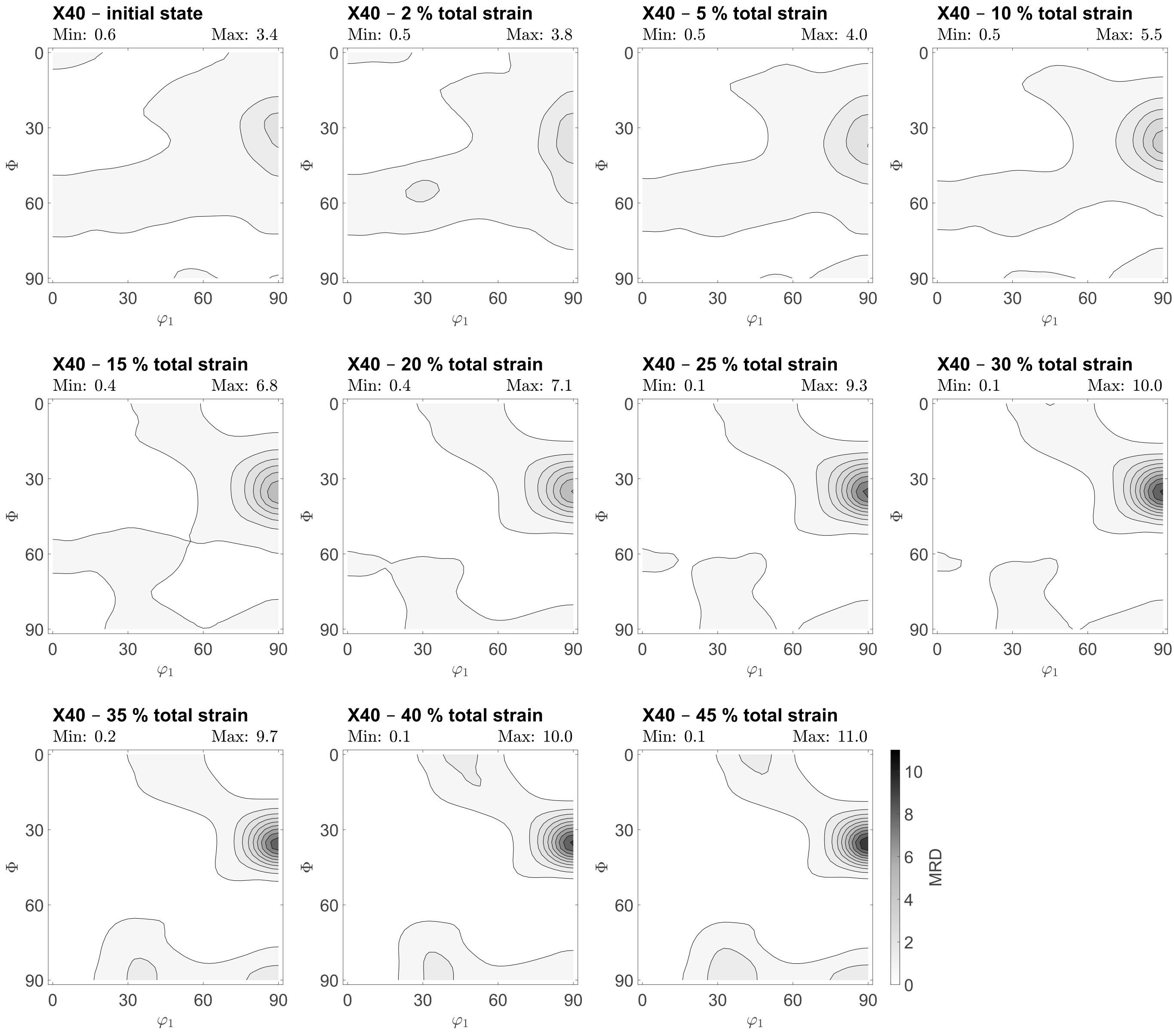

Figure 15: Texture evolution of steel X40MnCrAlV19-2.5-2 (X40) with the degree of deformation in rolling direction of the metal sheet. The cross section of the ODF at $\varphi_2 = 45$ ° is illustrated for all considered degrees of deformation. The displayed levels are steps of 1.0 × multiples of random distribution (MRD).

1.2 HCT690T (T7)

Figure 16 depicts the ODFs determined for the TRIP steel T7. It was deformed up to a total strain of approx. 25 %, so as not to exceed the range of uniform elongation.

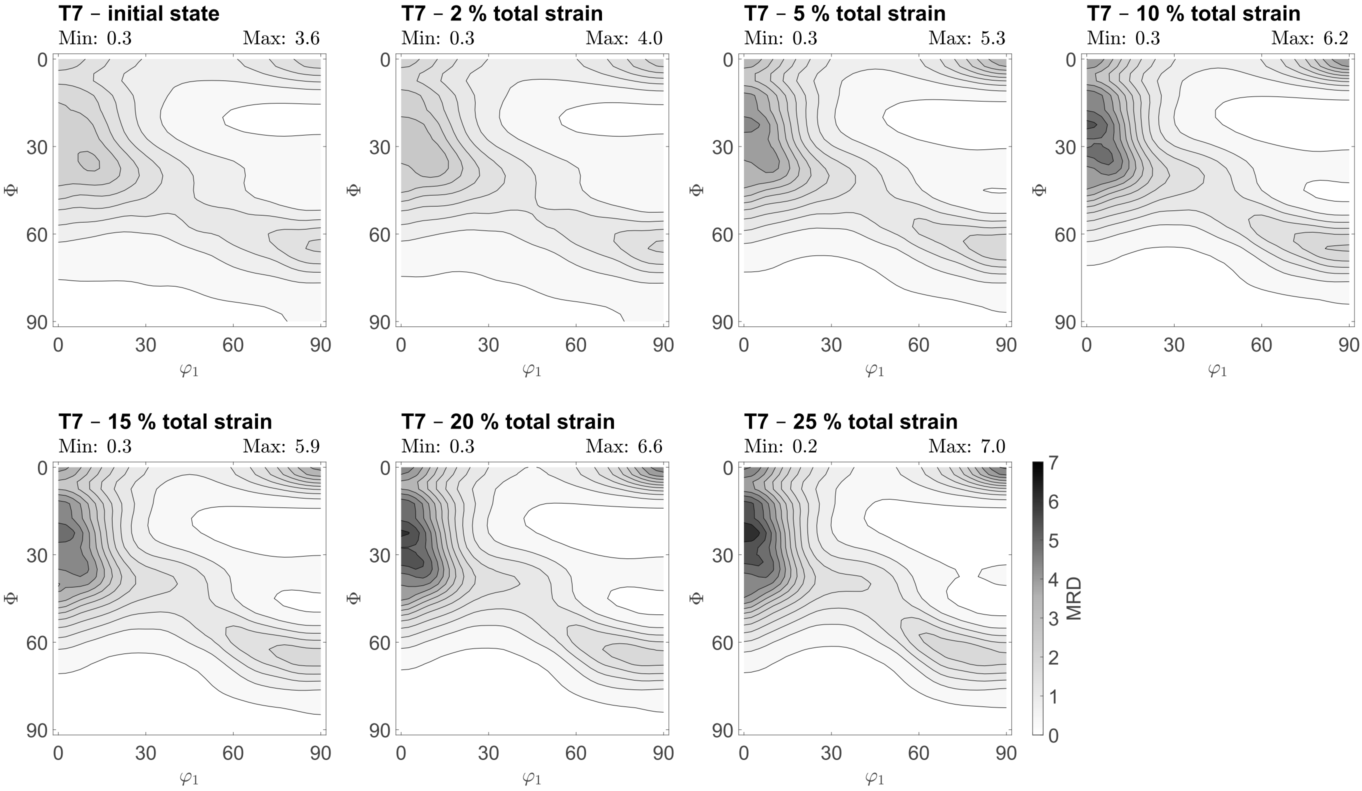

Figure 16: Texture evolution for the ferrite phase of HCT690T (T7) with the degree of deformation in the rolling direction of the metal sheet. The cross section of the ODF at $\varphi_2 = 45\ ^\circ$ is illustrated for all considered degrees of deformation. The displayed levels are steps of $1.0\ \times$ multiples of random distribution (MRD).

1.3 S355MC (S3)

Figure 17 depicts the ODFs determined for the structural steel S3. It was deformed up to a total strain of approx. 20 %, so as not to exceed the range of uniform elongation.

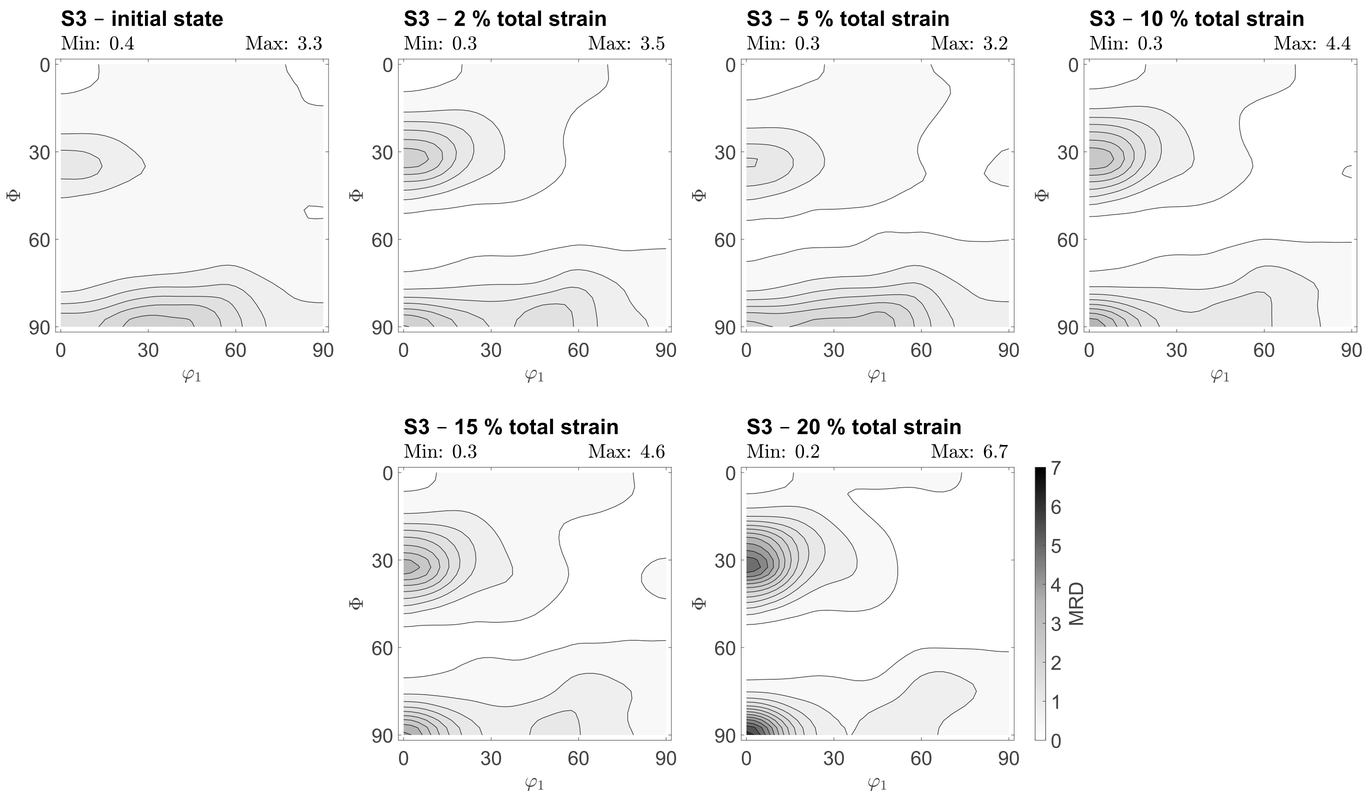

Figure 17: Texture evolution of steel S355MC (S3) with the degree of deformation in the rolling direction of the metal sheet. The cross section of the ODF at $\varphi_2 = 45$ ° is illustrated for all considered degrees of deformation. The displayed levels are steps of 0.5 × multiples of random distribution (MRD).

2 Quantification of texture components

2.1 Calculation method

The texture components and their respective volume fractions were determined using the open source Matlab MTEX toolbox (Bachmann *et al.*, 2010). The calculation of the volume fractions was based on a specified list of preferred orientations and a maximum misorientation angle $\Delta = 15$ °. On the one hand, this type of calculation means that the sum of the volume fractions of the texture components usually does not equal 100 %, since not the entirety of the orientation space is considered. On the other hand, the proportions of different components can overlap if their angular discrepancy is low, leading to an overestimation of their volume fractions. Therefore, the normalized intensity distributions determined for each component are also quantified using the multiple of random distribution (MRD). These distributions are not affected by this phenomenon, because they are derived from the orientations of the ideal components and are therefore independent of the chosen Δ range. Although other approaches might be better suited to differentiate between close texture components, the chosen method was used in this case, as it allows for a finer resolution

of texture components at overall low MRD values. Thus, texture components could be identified as present as soon as their MRD exceeded a self-defined threshold value of 1.5. The MRD values are always listed with the components in the following of this publication to distinguish between volume fraction and sharpness of a texture component.

2.2 X40MnCrAlV19-2.5-2 (X40)

The γ-fiber $\{111\} \parallel ND$ and the copper component $\{112\}\langle 111\rangle$ can be determined for the initial state (as-delivered) of steel X40. Here, the γ-fiber is regarded as the sum of components E and F, which are addressed in table 4. Cold rolling of austenitic steels can lead to the formation of a γ-fiber (Haase & Barrales-Mora, 2018; Tewary *et al.*, 2014; Barbier *et al.*, 2009; Vercammen *et al.*, 2004; Lü *et al.*, 2011). This processing combines strong plastic deformation and a plane strain condition, which results in mechanical twinning and reorientation of twin colonies to the rolling plane through coplanar slip as the result of an overshooting effect (Donadille *et al.*, 1989; Paul *et al.*, 2004). In contrast, plastic deformation, based primarily on dislocation slip, eventually leads to the formation of the copper component (Dillamore & Roberts, 1964). The copper component is a typical deformation texture of fcc materials and is also expected in the case of rolled sheets (Wassermann & Grewen, 1962; Bunge, 1969; Kocks *et al.*, 2000). Throughout the different degrees of uniaxial deformation, the copper component remains dominant. Instead of the γ-fiber, the Goss component $\{110\}\langle 111\rangle$, the cube component $\{001\}\langle 010\rangle$, and the A component $\{110\}\langle 111\rangle$ become more pronounced in the deformed state, in addition to the strong copper component. This is also confirmed in figure 18 (a), which shows the calculated volume fractions of the detected texture components. The volume fractions of the present texture components are low and the component-specific MRD values, depicted in figure 18 (b), emphasize that most of the examined components are close to the detection threshold. The copper component remains the strongest component throughout the deformation process, with its MRD increasing from approx.$\tilde{3}$.1 for the initial state to about 11.0 for the deformed state with about 45 % of total strain. The volume fraction of the copper component increases gradually from about 16 % to 41 %. The increase in the fraction of the copper component is the result of severe dislocation slip and is generally not expected to be the dominant texture for alloys with low stacking-fault energy, such as TWIP steels, which normally promote the formation of a $\{110\}\langle 112\rangle$ texture through twinning (Saleh *et al.*, 2014). However, *Dillamore* and *Roberts* suggest that intense deformation can lead to a change in the texture characteristics of a metal, partly based on the facilitation of cross slip caused by intergranular constraints within polycrystalline materials (Dillamore & Roberts, 1964). The complete lack of a copper twin component $\{552\}\langle 115\rangle$ also indicates slip-dominated deformation (Haase *et al.*, 2014). The other texture components exhibit volume fractions below about 22 % and MRD values below approx. 2.9. The previously mentioned γ-fiber can be detected for the initial state. Although the γ-fiber has MRD values below about 2.3, its spread within the orientation space leads to a comparably high volume fraction of approx. 21 % prior to uniaxial deformation. With the progression of uniaxial plastic deformation of the samples, the γ-fiber slowly loses significance.

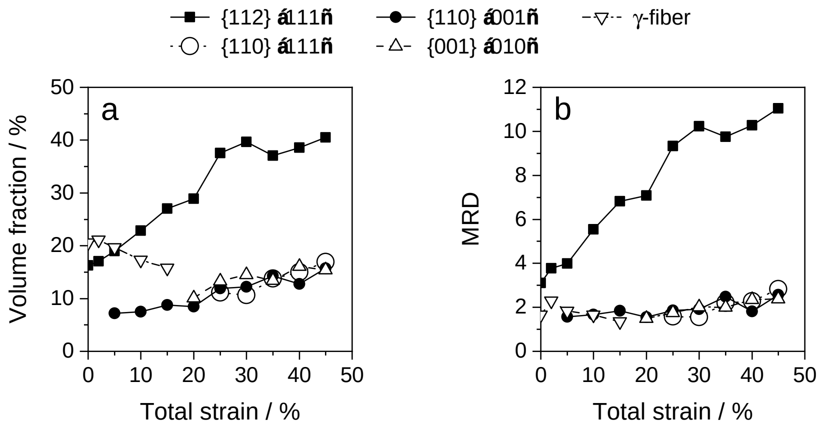

Figure 18: Quantification of the dominant texture components for steel X40MnCrAlV19-2.5-2 (X40) and their evolutions depending on the degree of deformation in rolling direction of the metal sheet. Their respective (a) volume fractions and (b) multiples of random distribution (MRD) are given for up to eleven degrees of deformation.

At a total strain of 20 %, it finally drops below the detection threshold. This decrease is likely due to the different load conditions of the rolling process and uniaxial deformation. While the plane strain condition of the rolling process leads to a uniform orientation of the sub grains due to twinning, the twins are expected to rotate the sub grains in many directions in the case of uniaxial deformation. Therefore, progressive uniaxial deformation must lead to a continuous decrease in the importance of the γ-fiber. However, a series of other components become evaluable with increasing deformation. In sequence, the Goss component is detectable from a total strain of approx. 5 %, the cube component from about 20 % and the A component from about 25 %. Their volume fractions and MRD values show the same trend. The A component is expected to be induced by dislocation slip, which is known to form during uniaxial deformation of fcc materials (Chin *et al.*, 1969; Savoie *et al.*, 1996; Chowdhury *et al.*, 2005). The evolution of the cube component after significant plastic deformation indicates cube-plane slip, i.e., slip on the {100} planes. It is the result of a low activation energy for dislocation slip (Dillamore & Roberts, 1964), which is consistent with the theory formulated for the amplification of the copper component.

2.3 HCT690T (T7)

Figure 19 shows the deformation-dependent volume fractions and MRD values determined for T7 metal sheets. In this case, however, it should be noted that the texture components of the α-fiber have similar angles in the orientation space. Therefore, the components overlap, leading to an overestimation of their respective volume fractions, as described in the supplementary section 2.1. As a consequence, their volume fractions are nearly identical and may only be considered in qualitative terms. The volume fraction of the transformed copper components increases by approx. 70 % and its MRD increases by about 3.2 when comparing the initial state to the state deformed to a

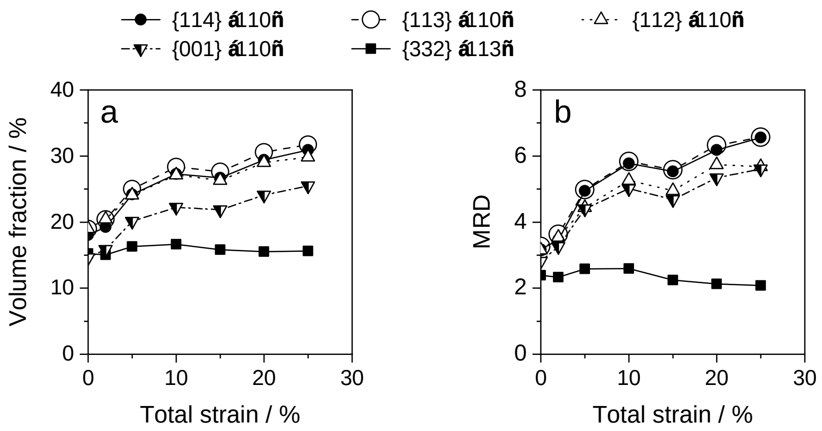

Figure 19: Quantification of the dominant texture components for steel HCT690T (T7) and their evolutions depending on the degree of deformation in rolling direction of the metal sheet. Their respective (a) volume fractions and (b) multiples of random distribution (MRD) are given for seven degrees of deformation.

total strain of 25 %. The intensity of the texture components increases significantly up to a total strain of 10 %. Afterwards, the rate of increase is declining. Instead of concentrating on individual copper components, a pronounced α-fiber (bcc) can also be adopted, especially since the present rotated cube component $\{001\}\langle110\rangle$ can also be seen as part of the α-fiber (bcc). As illustrated in figure 16, the rotated cube component is present at the positions $\varphi_1 = 0$ ° and $\varphi_1 = 90$ ° of the initial state, and its intensity increases during further uniaxial deformation. Likewise, the assumed α-fiber (bcc) of steel T7 is strongly pronounced for $\Phi < 60$ °. The transformed brass component $\{332\}\langle113\rangle$ is the only detectable texture component which is entirely independent of the α-fiber (bcc). It is well above the detection threshold, but its intensity is stagnating throughout the deformation experiment. Volume fraction and MRD of the transformed brass component vary by less than 1 % and 0.2, respectively, which is within the range of the measurement uncertainty. This behavior is expected since the $\{332\}\langle113\rangle$ texture originates from the rolling of the metal sheet. At the beginning of the rolling process, the austenite phase fraction is significantly higher than in its as-delivered state, and the deformation of the austenite phase leads to the formation of strong copper $\{112\}\langle111\rangle_\gamma$ and brass $\{110\}\langle112\rangle_\gamma$ components. After the phase transformation to ferrite, the said components are converted to $\{113\}\langle110\rangle_\alpha$ and $\{332\}\langle113\rangle_\alpha$ textures, respectively (Ray & Jonas, 1990). In contrast, the transformed copper component $\{113\}\langle110\rangle$ increases its intensity as part of the α-fiber (bcc), following further uniaxial deformation. Primary and conjugate slip are known to promote the evolution of the copper components, while cross slip enforces the rotated cube component $\{001\}\langle110\rangle$. A combination of both deformation mechanisms leads to an increasing MRD of the complete α-fiber (bcc) between these components, ranging between 0 ° $\leq \Phi \leq 35.3$ ° in the ODF shown in figure 16.

2.4 S355MC (S3)

For all investigated degrees of deformation, the sheets of steel S3 show the transformed copper component {112}⟨110⟩, the rotated Goss component {110}⟨110⟩, the A component {112}⟨111⟩, and the brass component {110}⟨112⟩. The existence of the rotated Goss component and its increasing intensity indicate the formation of shear bands accompanied by geometric softening phenomena (Nguyen-Minh *et al.*, 2015; Jain *et al.*, 2022). In fact, the S355MC shows a significant formation of Lüders bands during tensile deformation (Samek *et al.*, 2008). The slight brass texture in the as-delivered state is neither energetically nor geometrically favored to rotate or intensify due to the uniaxial tensile deformation and therefore shows no significant changes. Figure 20 shows that the volume fraction and MRD values of the transformed copper component and the rotated Goss component increase depending on the degree of deformation. Their volume fraction approx. doubles and the MRD value almost triples compared to the initial state. At the same time, the intensities of the A and brass components decrease continuously. At the final deformation of 20 % of total strain, said components are close to the detection threshold.

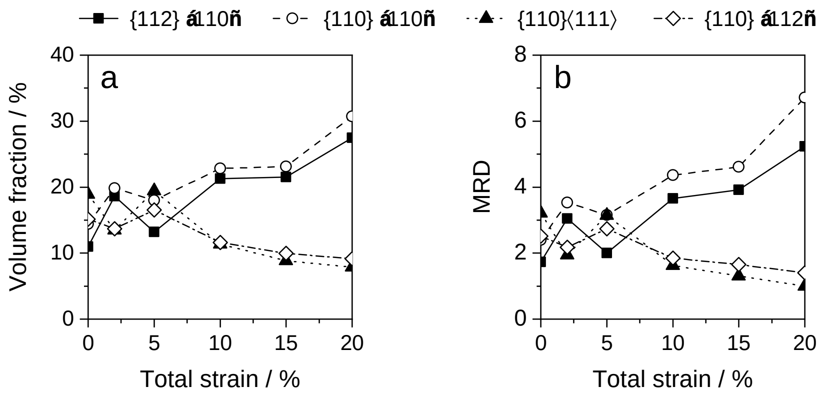

Figure 20: Quantification of the dominant texture components for steel S355MC (S3) and their evolutions depending on the degree of deformation in rolling direction of the metal sheet. Their respective (a) volume fractions and (b) multiples of random distribution (MRD) are given for six degrees of deformation.

References

Bachmann, F., Hielscher, R. & Schaeben, H. (2010). *Solid State Phenomena*, **160**, 63–68.

Barbier, D., Gey, N., Allain, S., Bozzolo, N. & Humbert, M. (2009). *Materials Science and Engineering: A*, **500**(1-2), 196–206.

Bunge, H.-J. (1969). *Texture Analysis in Materials Science*. Butterworths.

Chin, G. Y., Hosford, W. F. & Mendorf, D. R. (1969). *Proc. Roy. Soc. A*, **309**(1499), 433–456.

Chowdhury, S. G., Das, S. & De, P. K. (2005). *Acta Materialia*, **53**(14), 3951–3959.

Dillamore, I. L. & Roberts, W. T. (1964). *Acta Metallurgica*, **12**(3), 281–293.

Donadille, C., Valle, R., Dervin, P. & Penelle, R. (1989). *Acta Metallurgica*, **37**(6), 1547–1571.

Haase, C. & Barrales-Mora, L. A. (2018). *Acta Materialia*, **150**, 88–103.

Haase, C., Barrales-Mora, L. A., Roters, F., Molodov, D. A. & Gottstein, G. (2014). *Acta Materialia*, **80**, 327–340.

Jain, V., Modak, P., Patra, S. & Ghosh, A. (2022). *Materialia*, **22**.

Kocks, U. F., Tomé, C. N., Wenk, H.-R., Beaudoin, A. J. & Mecking, H. (eds.) (2000). *Texture and anisotropy: Preferred orientations in polycrystals and their effect on materials properties.* Cambridge: Cambridge University Press, first paperback edition (with corrections) ed.

Lü, Y., Molodov, D. A. & Gottstein, G. (2011). *ISIJ International*, **51**(5), 812–817.

Nguyen-Minh, T., Sidor, J. J., Petrov, R. H. & Kestens, L. A. I. (2015). *IOP Conference Series: Materials Science and Engineering*, **82**.

Paul, H., Morawiec, A., Piatkowski, A., Bouzy, E. & Fundenberger, J. J. (2004). *Metallurgical and Materials Transactions A*, **12**, 3775–3786.

Ray, R. K. & Jonas, J. J. (1990). *International Materials Reviews*, **35**(1), 1–36.

Saleh, A. A., Haase, C., Pereloma, E. V., Molodov, D. A. & Gazder, A. A. (2014). *Acta Materialia*, **70**, 259–271.

Samek, L., De Moor, E., Penning, J., Speer, J. G. & De Cooman, B. C. (2008). *Metallurgical and Materials Transactions A*, **39**(11), 2542–2554.

Savoie, J., Zhou, Y., Jonas, J. J. & Macewen, S. R. (1996). *Acta Materialia*, **44**(2), 587–605.

Tewary, N. K., Ghosh, S. K., Bera, S., Chakrabarti, D. & Chatterjee, S. (2014). *Materials Science and Engineering: A*, **615**, 405–415.

Vercammen, S., Blanpain, B., De Cooman, B. C. & Wollants, P. (2004). *Acta Materialia*, **52**(7), 2005–2012.

Wassermann, G. & Grewen, J. (1962). *Texturen metallischer Werkstoffe.* Springer.
